# Research on epicardial adipose tissue as a metabolic therapeutic target in AF: focus on GLP-1 receptor agonists

**DOI:** 10.3389/fcvm.2026.1708109

**Published:** 2026-03-05

**Authors:** Ling Liu, Junlin Lu

**Affiliations:** 1 Department of Nursing, Chongqing Rongchang Hospital of Traditional Chinese Medicine, Chongqing, China; 2 Department of Medical Affairs, Chongqing Rongchang Hospital of Traditional Chinese Medicine, Chongqing, China

**Keywords:** atrial fibrillation, atrial remodeling, epicardial adipose tissue, glucagon-like peptide-1 receptor agonist, therapeutic target

## Abstract

Epicardial adipose tissue (EAT), a metabolically active visceral fat depot anatomically contiguous with the myocardium, has emerged as a critical mediator and promising metabolic therapeutic target in atrial fibrillation (AF), particularly in the context of obesity and diabetes mellitus. Pathological expansion and dysfunction of EAT promote AF through paracrine and vasocrine secretion of pro-inflammatory and pro-fibrotic cytokines, release of extracellular vesicles carrying arrhythmogenic cargo, direct infiltration, and modulation of local electrophysiology and autonomic signaling, thereby creating a substrate for atrial cardiomyopathy, fibrosis, electrical remodeling, and AF initiation/persistence. Glucagon-like peptide-1 receptor agonists (GLP-1RAs), beyond their glucoregulatory and weight-loss benefits, exhibit potential cardioprotective effects that may be relevant to AF. Notably, GLP-1 receptors are expressed in both human EAT and atrial tissue. Preclinical and clinical studies suggest that GLP-1RAs may reduce EAT volume/thickness, potentially exceeding overall weight loss, attenuate EAT inflammation and fibrosis, improve cardiomyocyte calcium handling, mitigate oxidative stress, and suppress pulmonary vein ectopy, thereby potentially reducing AF susceptibility and recurrence post-ablation. While evidence from genetic studies, meta-analyses, and specific cardiovascular outcome trials (CVOTs) supports an association between certain GLP-1RAs and reduced AF risk, conflicting data exist regarding drug-specific effects, underscoring the need for further mechanistic and outcome research. Targeting EAT modulation via GLP-1RAs represents a compelling strategy to disrupt the obesity–diabetes–AF axis, although challenges remain in elucidating precise molecular mechanisms, standardizing EAT assessment, understanding response heterogeneity, and defining the clinical role of specific GLP-1RAs within AF management algorithms.

## Graphical Abstract

**Figure:**
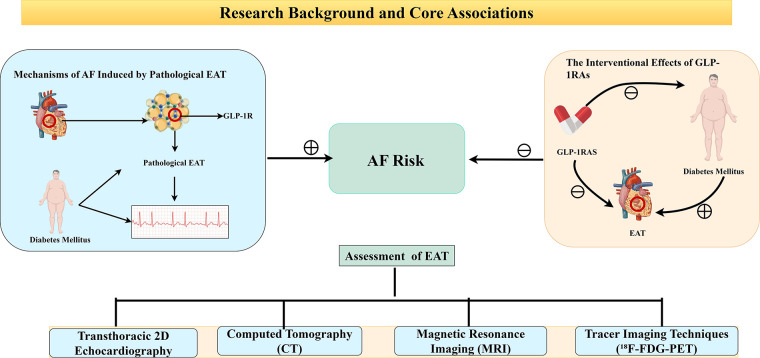
Illustrations from BioGDP (https://biogdp.com/help).

## 1 Introduction

Atrial fibrillation (AF) is the most prevalent sustained cardiac arrhythmia globally, affecting approximately 50 million individuals worldwide. Its cardinal pathological hallmark is perturbation of atrial electrical activity, with clinical manifestations including palpitations, dyspnea, and other associated symptoms. Beyond compromising quality of life, AF notably augments the risks of adverse clinical outcomes and mortality by predisposing patients to complications such as thromboembolism and heart failure (1, 2). As a well-established independent risk factor for cardiovascular disorders, diabetes mellitus (DM) affects roughly 38.4 million adults in the United States (3) and is associated with a marked increase in the risk of developing AF and atrial flutter (AFL) (4). Furthermore, owing to the presence of shared risk determinants including obesity and hypertension, these two conditions frequently coexist (5, 6). Accumulating evidence confirms that the prevalence of AF among diabetic patients is twice that observed in non-diabetic cohorts (7). Compared with patients diagnosed with diabetes alone, those with diabetes complicated by AF exhibit substantially higher risks of thromboembolic events (e.g., ischemic stroke) and cardiovascular-related mortality (1, 8). In particular, patients with type 2 diabetes mellitus (T2DM) have a 35% higher risk of developing AF (9). Type 1 diabetes mellitus (T1DM) is also independently linked to an increased incidence of AF; according to data retrieved from the Swedish National Diabetes Register, the adjusted hazards ratio (HR) for AF in T1DM patients is 1.13 (95% CI 1.01–1.25, *P* = 0.029) in men and 1.50 (95% CI 1.30–1.72, *P* < 0.0001) in women (10). Bisson et al. similarly demonstrated that the adjusted HR for AF is higher in female diabetic patients than in their male counterparts (17% vs. 10%). Plausible explanations for this phenomenon include impaired physiological protective mechanisms, suboptimal control of risk factors, and gender-specific susceptibility to metabolic and inflammatory pathways in women with diabetes. Furthermore, this gender discrepancy persists across all age strata in T1DM, whereas it is confined to middle age in T2DM (11). In addition, obesity constitutes a significant risk factor for AF; global prevalence of obesity has tripled since 1975, and its status as an independent risk factor for AF is well-established (12–15). Current clinical guidelines designate weight loss as a Class Ⅰ indication for AF management in patients with a body mass index (BMI) > 27 kg/m^2^ (2).

However, the specific mechanisms underlying obesity-induced AF remain incompletely elucidated. Accumulating evidence suggests that epicardial adipose tissue (EAT) may serve as a key mediator linking obesity to AF. As a distinct visceral fat depot, EAT is enriched in genes involved in endothelial function, coagulation, immune signaling, lipid metabolism, potassium ion transport, and apoptosis (16). EAT contributes to the pathogenesis and progression of AF, with a particularly high prevalence in obese populations. Clinically, EAT holds broad applications in cardiovascular medicine, spanning from imaging quantifiability to drug responsiveness. It is a highly inflammatory fat reservoir characterized by a pro-inflammatory and pro-fibrotic transcriptome. Compared with subcutaneous adipose tissue and other visceral adipose depots, EAT exhibits the most pronounced inflammatory properties (17). Emerging studies have demonstrated that abnormal EAT, as a source of pro-inflammatory and fibrotic mediators, induces atrial myopathy by exerting paracrine effects on the adjacent atrial myocardium, thereby generating an arrhythmogenic substrate necessary for the initiation and maintenance of AF (18–21). Given its metabolic activity, organ-specific fat distribution, and ease of measurement, EAT represents a modifiable therapeutic target for adipose tissue-modulating agents, such as glucagon-like peptide-1 receptor agonists (GLP-1RAs) (22).

Accumulating clinical evidence indicates that, beyond glycemic control in diabetes, GLP-1RAs exert weight-reducing and cardioprotective effects (23, 24) and can modestly decrease the thickness of EAT (25–27). Iacobellis et al. were the first to validate the expression of glucagon-like peptide-1 receptors (GLP-1Rs) in human EAT at both the mRNA and immunofluorescence levels. This finding suggests that the favorable cardiovascular effects of GLP-1RAs may be mediated, at least in part, through direct targeting of this specific fat depot, although the precise cell types involved remain undefined (28). Concurrently, research has revealed that GLP-1R is highly expressed in atrial tissues, suggesting that GLP-1 may participate in atrial remodeling processes (29). Taken together with evidence that EAT expresses GLP-1R and that GLP-1RAs can reduce EAT volume, this finding further indicates that GLP-1RAs may have the potential to mitigate AF risk (26). However, current literature regarding the association between GLP-1RA therapy and AF risk remains controversial.

Rhythm control strategies (e.g., direct current cardioversion, antiarrhythmic agents, and catheter ablation) are central to maintaining sinus rhythm (SR), alleviating symptoms, and improving quality of life in patients with AF. Among these modalities, catheter ablation is the most effective rhythm control therapy for symptomatic AF patients who are refractory to pharmacological treatment (30, 31). However, accumulating evidence indicates that the success rate of ablation is reduced by up to 10% in obese individuals (BMI ≥ 30) (32). Furthermore, when AF coexists with DM, the risk of thromboembolic events is significantly elevated, yet current clinical practice guidelines offer limited guidance on therapeutic strategies for patients with comorbid AF and DM. These limitations underscore an urgent need for therapeutic approaches that not only achieve weight reduction but also exert a therapeutic effect on both AF and DM. Based on this premise, the present study aims to review the potential of GLP-1RAs as a metabolic therapeutic target for AF via modulation of EAT.

## 2 Mechanism of atrial fibrillation

To date, the pathogenesis of AF remains incompletely understood and is believed to primarily involve the following aspects:

Myocardial metabolic dysfunction: (1) During AF, myocardial metabolism undergoes marked alterations, characterized by reduced efficiency of fatty acid oxidation. To compensate, energy metabolism shifts toward a glucose-dominated “embryonic metabolic pattern” (33). (2) Calcium/calmodulin-dependent protein kinase II (CaMKII) and adenosine monophosphate-activated protein kinase are upregulated, and the expression of fatty acid translocase (FAT/CD36) is increased. This imbalance in fatty acid metabolism can accelerate AF progression (34, 35). (3) In patients with AF, myocardial glucose uptake is decreased while metabolic activity is enhanced, accompanied by increased glycolytic activity and impaired mitochondrial function. An imbalance between the production and clearance of reactive oxygen species (ROS) further contributes to alterations in cardiomyocyte morphology and function (36–39).
Atrial remodeling: (1) Structural remodeling: Multiple factors, including inflammatory responses, paracrine/vasocrine effects of EAT, and elevated chronic hemodynamic stress, can activate atrial fibroblasts, promote their proliferation and differentiation, alter the expression of relevant proteins and cytokines, and ultimately drive the pathogenesis and progression of atrial fibrosis (40–45). (2) Electrical remodeling: Inflammatory mediators alter atrial electrophysiological properties and structural substrates, disrupt calcium homeostasis and connexins, and trigger abnormal atrial conduction (46, 47). Enhanced ganglion plexus (GP) activity within EAT augments the modulatory effect of the autonomic nervous system (ANS) on atrial cardiomyocytes and shortens action potential duration (APD) (48). Concurrently, atrial fibrosis disrupts normal electrical conduction pathways, leading to conduction slowing or block and providing a substrate for the generation and maintenance of abnormal electrical impulses (45).
Autonomic nervous system imbalance: The interplay between the sympathetic and parasympathetic nervous systems is critical to the initiation and maintenance of AF. In some patients, AF is linked to excessive vagal activity, typically occurring at night and in the presence of a smaller left atrial (LA) volume; in others, it is triggered by sympathetic activation, predominantly during strenuous exercise or emotional stress (49, 50).
Genetic factors: To date, over 160 AF-associated genes have been identified. Genome-wide association studies have identified more than 260 single-nucleotide polymorphisms linked to AF pathogenesis, and genetic risk scores may enhance the accuracy of AF risk stratification (51, 52).
Inflammasome activation: Persistent activation of the nucleotide-binding oligomerization domain-like receptor family pyrin domain-containing 3 (NLRP3) inflammasome is strongly linked to AF. By inducing inflammatory responses, it can lead to atrial hypertrophy, shorten the effective refractory period, and cause abnormal calcium release, thereby facilitating the initiation and progression of AF. Conversely, inhibiting NLRP3 expression can suppress AF development (53, 54).

## 3 Pathogenic mechanisms underlying DM with concurrent AF

The mechanisms underlying the increased susceptibility to AF/AFL in patients with DM remain elusive. Previous studies have indicated that the association between DM and AF may involve mechanisms such as oxidative stress, inflammatory responses, abnormal energy metabolism, impaired cellular calcium handling, dysfunctional ion channels, and conduction disturbances (55). Specifically, the underlying mechanisms may include the following: (1) Advanced glycation end products (AGEs) exert their effects by binding to the receptors for advanced glycation end products (RAGEs), which upregulates the production of pro-inflammatory mediators, facilitates protein cross-linking, and ultimately exacerbates atrial structural remodeling (56). (2) Activation of the AGE–RAGE pathway induces ROS generation, which, in combination with mitochondrial dysfunction under diabetic conditions, exacerbates myocardial injury, further accelerates atrial remodeling, and promotes AF development (57, 58). (3) Patients with DM frequently present with metabolic syndrome, which is characterized by hyperglycemia, hypertension, and obesity. Moreover, fluctuations in hyperglycemia can promote AF development by inducing myocardial structural, electromechanical, and autonomic nervous system remodeling (59, 60). (4) DM-associated chronic inflammatory responses further contribute to atrial remodeling and enhance susceptibility to AF (61).

## 4 EAT

### 4.1 Development, distribution, and function of EAT

Adipose tissue surrounding the heart can be categorized into pericardial adipose tissue and epicardial adipose tissue. The former is located between the pericardial leaflets (62), whereas the latter is predominantly distributed along the atrioventricular and interventricular grooves. Epicardial adipose tissue can be further subdivided into perivascular epicardial adipose tissue (directly adjacent to the coronary artery adventitia) and myocardial epicardial adipose tissue (directly overlying the myocardial surface) (63).

Visceral fat assessment is an effective approach for identifying high-risk populations and mitigating cardiovascular risks. As a specialized visceral fat depot, EAT possesses distinct anatomical, functional, and genetic characteristics. Its volume is significantly elevated in patients with T2DM and metabolic syndrome and appears to be independent of conventional body fat metrics (64–66). Compared with other visceral fat depots, EAT exhibits greater metabolic activity. On a per-gram basis, EAT contains smaller yet more numerous adipocytes, characterized by lower glucose utilization but higher rates of fatty acid uptake and release (67). In healthy individuals, EAT constitutes approximately 20% of total cardiac weight and covers nearly 80% of the cardiac surface area (68). In contrast, patients with impaired fasting glucose, insulin resistance, or T2DM exhibit significantly increased EAT thickness, volume, and surface area, particularly along the right ventricular free wall (69). Expansion of EAT is primarily mediated by adipocyte hyperplasia (the formation of new adipocytes), which contrasts with the expansion of other visceral and subcutaneous fat depots, where cell hypertrophy (increased cell volume) predominates (70–72). Consequently, adipocyte size within EAT remains unchanged or increases only slightly despite volumetric expansion (73). Factors including age, gender, race, and BMI influence the distribution and thickness of EAT (74). Studies have demonstrated that EAT thickness increases by approximately 22% by 65 years of age compared with baseline levels (75).

In recent years, EAT has garnered extensive attention as a key risk factor for cardiovascular disease and a potential therapeutic target. EAT arises from the visceral pleural mesoderm and functions as a metabolically active fat depot situated between the myocardium and visceral pericardium (63). Its cellular components extend beyond adipocytes to include nerve cells, inflammatory cells (e.g., macrophages, mast cells), stromal cells, vascular cells, and other immune cells (63). Functionally, EAT exhibits properties of white adipose tissue while also displaying brown and beige adipose-like features; however, its brown adipose-like traits are progressively downregulated with aging and the progression of end-stage chronic diseases (27). During the neonatal period and under conditions of hemodynamic abnormalities such as ischemia and hypoxia, EAT can protect the myocardium via direct thermogenesis (76). Beyond its thermogenic role, EAT supports myocardial energy demands by releasing and uptaking free fatty acids (FFAs) (with FFA oxidation contributing 50%–70% of myocardial energy production). Notably, fatty acid-binding protein 4 is highly expressed in EAT and participates in FFA metabolism. Moreover, EAT buffers lipotoxicity between the myocardium and local vasculature by chelating excess FFAs, a function that is critical for maintaining myocardial health under metabolic stress (77).

EAT surrounds the coronary arteries and lacks a myofascial barrier separating it from the myocardium, thereby sharing a blood supply with the deep myocardium. Given its anatomical proximity to the coronary arteries, EAT is likely to exert a significant impact on myocardial microvascular function. Concurrently, EAT secretes a wide spectrum of pro-inflammatory and anti-inflammatory adipocytokines, which can contribute to microvascular obstruction via paracrine and vasocrine mechanisms (78–81). Owing to its distinctive distribution features, paracrine functions, and metabolic traits, EAT has garnered extensive attention as an imaging biomarker for metabolic and cardiovascular diseases, serving as a “transducer” that conveys the cardiotoxic effects induced by chronic inflammation and metabolic disturbances (18). Research indicates that excessively hyperplastic and dysfunctional EAT exerts adverse cardiac effects primarily through two pathways: first, by serving as a source of pro-inflammatory mediators, and second, through direct cardiac mechanical effects, ultimately promoting cardiovascular disease (82).

Compared with subcutaneous fat, EAT is highly enriched in genes associated with inflammatory, coagulative, and immune signaling pathways and exhibits high expression of brown adipocyte-specific proteins such as uncoupling protein 1 (83). In the obese state, EAT expansion induces a pro-inflammatory and pro-oxidative cardiac microenvironment, promotes ectopic infiltration of cardiomyocytes, and thereby contributes to the development of insulin resistance, endothelial dysfunction, mitochondrial dysfunction, and myocardial fibrosis (63). It is also linked to diabetes (84), atherosclerosis (85), coronary artery disease (86), and atrial fibrillation (87).

### 4.2 Methods for assessing EAT

Precise assessment of EAT provides a crucial basis for risk stratification and prognostic evaluation of AF and AFL, particularly in high-risk obese populations. Currently, common assessment modalities in clinical practice and research exhibit distinct characteristics, with their core technologies and application values outlined as follows.

Echocardiography: Standard two-dimensional transthoracic echocardiography is a widely used, minimally invasive modality for EAT visualization and thickness measurement. Parasternal long-axis and short-axis views are the preferred imaging planes. EAT appears as a hypoechoic region between the outer myocardial wall and the visceral pericardium, with a reference thickness range of 1–25 mm. This technique offers advantages of high reproducibility, low cost, and ease of operation. However, it also has notable limitations: it only enables single-site thickness measurement, failing to reflect the heterogeneity of EAT distribution; it is prone to missing global thickness changes in severely obese populations; and it cannot accurately quantify volumetric parameters (76).

Computed tomography and magnetic resonance imaging: Both serve as core modalities for precise quantification of EAT, as both enable volumetric measurement and three-dimensional evaluation. Coronary computed tomography angiography (CCTA) can clearly capture perivascular EAT around the coronary arteries, acquire the fat attenuation index to reflect EAT density, and achieve automated and rapid quantification of EAT volume through the integration of machine learning and artificial intelligence technologies, thereby providing an optimized basis for patient risk stratification (88–90). Cardiac magnetic resonance imaging can further quantify the proportion of fatty acids in EAT, providing complementary information to CT imaging. Moreover, it exhibits high sensitivity and specificity in localizing EAT distribution, making it a precise modality for the quantitative assessment of EAT. However, both modalities are limited by higher cost and limited accessibility, and CCTA also involves exposure to ionizing radiation (91–94).

**Figure 1:**
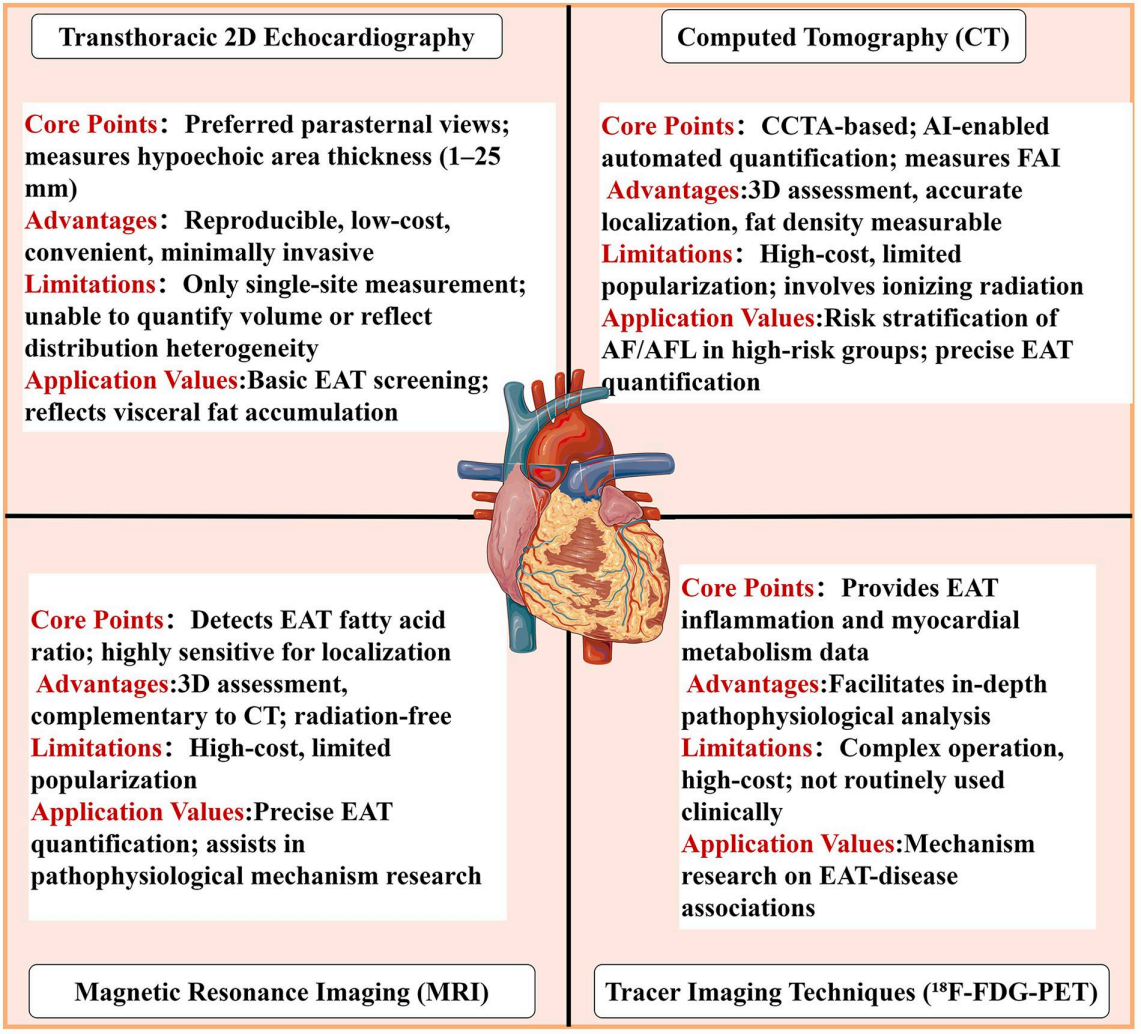
Methods for assessing EAT. Illustrations from BioGDP (https://biogdp.com/help).

Tracer imaging modalities: Techniques such as ^18^F-fluorodeoxyglucose positron emission tomography can provide information regarding the inflammatory activity of EAT and myocardial metabolism, thereby complementing the in-depth elucidation of EAT pathophysiology. However, due to their procedural complexity and high costs, these modalities are currently primarily utilized for mechanistic studies and have not been adopted for routine clinical evaluation (95) (Figure 1).

### 4.3 Relationship between EAT and AF

**Figure 2:**
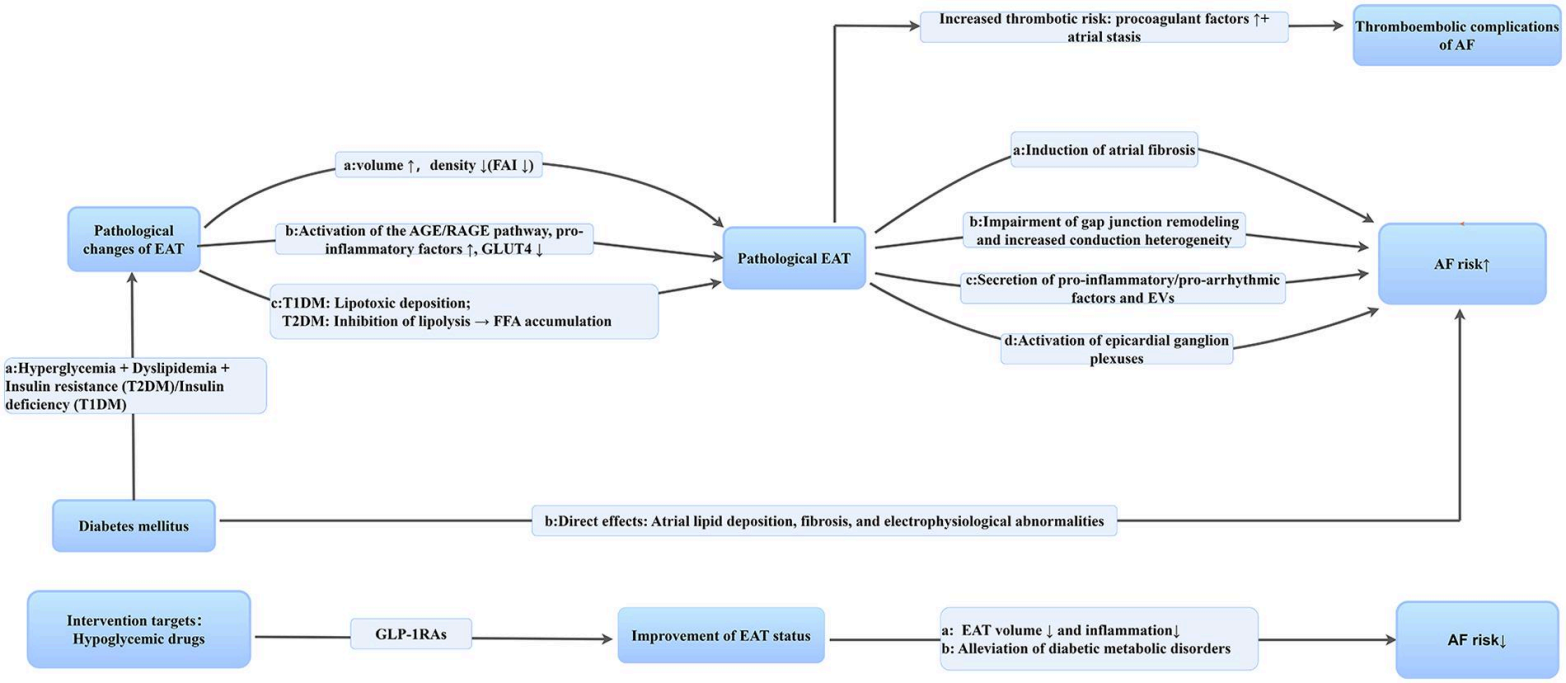
Possible relationship between AF, EAT, and GLP-1RAs.

EAT remodeling is strongly linked to AF and serves as one of the key anatomical substrates for the development of AF. Its association with AF may involve the following aspects (Figure 2).

EAT can interact with the left atrial myocardium through the secretion of adipokines, reactive oxygen species, and other substances, thereby influencing the onset and progression of AF. Huber et al. demonstrated using cCT that LA EAT dispersion—a metric reflecting EAT heterogeneity, defined as the difference between mean attenuation values of low- and high-threshold EAT compartments—was significantly greater in patients with persistent AF than in those with paroxysmal AF [52.6 HU vs. 49.9 HU (*p* = 0.001)]. Furthermore, patients with LA EAT dispersion above the mean value (>50.8 HU) exhibited a significantly elevated risk of AF recurrence within 1 year post-procedure [HR = 2.3, 95% CI: 1.5–3.6 (*p* < 0.001)]. This finding underscores the significance of EAT heterogeneity in AF progression and prognosis, thereby providing a rationale for its application as a therapeutic target for AF (96). In a separate study focusing on LA EAT mass (a parameter representing overall volume/weight), van Rosendael et al. observed that LA EAT mass was significantly higher in patients with paroxysmal AF than in those with SR [9.4 ± 5.4 g vs. 5.5 ± 3.7 g (*p* < 0.001)], whereas no significant difference was noted between patients with persistent/permanent AF and those with paroxysmal AF [10.4 ± 6.7 g vs. 9.4 ± 5.4 g (*p* = 0.563)]. Notably, when LA EAT mass was large but LA volume was small, patients were more likely to present with paroxysmal AF (55.2%) than with persistent/permanent AF (12.0%). This observation suggests that abnormal EAT mass may act as a driving factor in the early pathophysiological process of AF rather than a concomitant consequence of late LA structural remodeling (characterized by increased LA volume) (82). Collectively, these two studies elucidate the role of EAT in AF from two distinct dimensions: EAT “heterogeneity” (pathological complexity) and “overall mass” (early quantitative changes).

The role of EAT in AF may be rooted in its embryonic development. The embryonic epicardium is capable of generating coronary artery smooth muscle cells and cardiac fibroblasts, as well as retains the ability to undergo adipogenic differentiation (97). Atrial EAT arises from secretions of epicardial progenitor cells and atrial myocytes; in particular, atrial natriuretic factor released by atrial myocytes under mechanical stress stimulation exerts adipogenic effects, potentially promoting EAT formation (98).

As an endocrine organ, EAT supports cardiovascular health under normal physiological conditions by providing mechanical support, supplying energy, and secreting various adipokines. Under pathological conditions, however, excessive EAT accumulation releases pro-inflammatory adipokines via vasocrine or paracrine pathways, exacerbating cardiac damage (99), a phenomenon particularly prevalent in patients with coronary artery disease and diabetes mellitus (95). In patients undergoing cardiac surgery, EAT exhibits intense inflammation, fibrosis, angiogenesis, and apoptosis and can affect the adjacent myocardium through paracrine mechanisms or direct cellular infiltration (18–20). Furthermore, atrial-infiltrating EAT upregulates the expression of genes encoding proteins with potential arrhythmogenic properties, including those involved in oxidative phosphorylation, muscle contraction, and calcium signaling (100).

Abnormal EAT acts as a source of pro-inflammatory and fibrotic molecules that can impact adjacent atria, induce atrial myopathy, and generate arrhythmogenic substrates necessary for the initiation and maintenance of AF (18–21). These molecules can be packaged into extracellular vesicles (EVs)—membrane-bound organelles released by all cell types that are capable of local or distant transport and biological signaling (101, 102). EAT derived from AF patients has been shown to secrete abundant EVs, which accelerate the progression of atrial myopathy and AF due to their strong pro-inflammatory, pro-fibrotic, and arrhythmogenic properties (87). Furthermore, pro-inflammatory and pro-fibrotic cytokines, including activin A, visfatin, transforming growth factor-β1 (TGF-β1), and MCP-1, promote fibroblast proliferation and myofibroblast activation, resulting in atrial and ventricular arrhythmias (103).

EVs derived from adipose tissue are linked to obesity-related disorders, including metabolic syndrome, type 2 diabetes mellitus, and endothelial dysfunction (104). EAT-derived EVs can promote atrial fibrosis, impair intercellular conduction among cardiomyocytes, and induce local conduction blocks and conduction heterogeneity, thereby creating favorable conditions for arrhythmia reentry and persistence (105). Furthermore, inflammatory and fibrotic mediators can directly modify atrial electrophysiology and enhance AF susceptibility—for instance, by regulating calcium homeostasis and connexins, which are associated with AF triggers and slow atrial conduction (20, 106, 107). However, current EV isolation approaches are limited by issues such as protein contamination, challenges in verifying vesicle origin, and potential contamination from apoptotic cells and apoptotic bodies, which hinder EV research (87).

EAT can modulate local electrophysiological properties, such as the refractory periods of the atria and pulmonary veins, thereby sustaining AF. Studies have demonstrated that localized accumulation of EAT surrounding the atria—rather than overall cardiac adiposity—plays a pivotal role in inducing conduction abnormalities that lead to AF (20). Further research by Iacobellis et al. confirmed that left atrial EAT, through the production of pro-fibrotic and pro-inflammatory mediators, facilitates the formation of myocardial reentrant circuits and promotes the excessive release of free fatty acids (FFAs) into cardiomyocytes, impairing their continuity and prolonging the action potential (63). Furthermore, EAT volume has been shown to correlate positively with AF duration, independent of BMI or other risk factors (108). Notably, patients with metabolic syndrome or T2DM exhibit abnormal enlargement and biotransformation of EAT and experience higher relative event rates compared to obese individuals with lower EAT volume (109).

## 5 GLP-1RAS

### 5.1 Biological characteristics of GLP-1RAs and their cardiovascular action basis

The GLP-1R is expressed in the human pancreas, intestines, and heart, with distribution across all four cardiac chambers and predominant localization in the sinoatrial node (110). As the endogenous ligand of GLP-1R, GLP-1 is a peptide hormone secreted by pancreatic islets that selectively binds to and activates this receptor. Its hypoglycemic effect was initially identified due to the presence of GLP-1R on pancreatic *α*, *β*, and *δ* cells. Although the insulinotropic activity of GLP-1 was identified over 30 years ago, the short half-life of native GLP-1 (approximately 2 min)—attributed to rapid enzymatic degradation by dipeptidyl peptidase-IV—impeded its early therapeutic application in T2DM (111). To enhance its bioavailability and clinical efficacy, researchers therefore developed synthetic GLP-1 analogs, which in turn led to GLP-1RAs (112). GLP-1RAs are a novel class of glucose-lowering agents that mimic the physiological actions of endogenous GLP-1 by enhancing insulin secretion, reducing glucagon release, and acting on the brain to inhibit gastrointestinal motility, thereby slowing nutrient absorption. Furthermore, GLP-1RAs transmit signals via neuronal GLP-1R to suppress appetite, and their sustained administration of induces weight loss (113). Beyond glycemic control and weight reduction, GLP-1RAs confer cardiovascular, renal, and metabolic benefits, positioning them among the most promising therapeutic options, particularly in obese patients with diabetes (111). GLP-1-related signaling is mediated by GLP-1 binding to its specific receptors on diverse cell types (including pancreatic, intestinal, myocardial, central nervous system, and adipocytes), thereby regulating a wide range of physiological processes such as pancreatic secretion, appetite modulation, cardiovascular function, mood, behavior, and cognitive function (114, 115).

Clinical and preclinical evidence indicates that GLP-1RAs can improve myocardial metabolism—an effect that may in part account for their ability to reduce the incidence of AF (116). Accordingly, studies have further identified that non-glycemic mechanisms contribute to these cardioprotective benefits, including weight reduction, blood pressure lowering, anti-fibrotic activity, and improvements in microcirculatory endothelial function and conduction properties—all of which may mitigate the development of AF (117). Given that GLP-1RAs exert beneficial effects on risk factors for new-onset AF (e.g., body weight, blood pressure, renal function, and glycemic control), their impact on AF events has emerged as an area of growing interest in academia and clinical settings (118, 119). Currently, GLP-1RAs—including liraglutide, exenatide, dulaglutide, semaglutide, and albiglutide—have gained substantial attention in cardiovascular disease research, beyond their established role in glycemic management for patients with obesity and T2DM.

### 5.2 Studies on GLP-1RAs and AF

In animal studies, numerous previous experiments have demonstrated that GLP-1RAs exert ameliorative effects on AF. In a diabetic mouse model, liraglutide reduced AF incidence and prevented atrial remodeling in type 2 diabetes mellitus mice (29). Similarly, in a pacing-induced AF canine model, 3 weeks of liraglutide treatment resulted in alterations in electrophysiological parameters, with a markedly lower AF inducibility compared with controls (120). Regarding specific mechanisms, Wang et al. revealed that liraglutide inhibits angiotensin II (AngII)-induced cardiac fibroblast proliferation, migration, and extracellular matrix (ECM) deposition by regulating the miR-21/PTEN/PI3K signaling pathway, thereby mitigating myocardial fibrosis (121). Concurrently, liraglutide alleviates rapid atrial pacing-induced atrial electrical remodeling by suppressing reductions in conduction velocity (CV) and limiting increases in AF susceptibility, effects that are potentially linked to the regulation of calcium homeostasis and antioxidant stress (122).

In human studies, accumulating evidence supports an association between GLP-1RAs and a reduced risk of AF. A real-world study of American adults with diabetes demonstrated that GLP-1RA use was associated with a lower incidence of AF (123). Similarly, Zhang et al.'s research also indicated that genetically predisposed GLP-1Ras were correlated with decreased risks of AF, cardiac arrest, and ventricular fibrillation (124). Meta-analyses further revealed that in individuals at high cardiovascular risk, semaglutide reduced AF episodes by 42% (125). Notably, another meta-analysis of 21 randomized controlled trials (RCTs) involving 25,957 patients with T2DM, obesity, or overweight demonstrated that semaglutide was linked to a lower risk of AF incidence (126). In addition, studies have found that compared with placebo, semaglutide significantly reduced AF incidence by 42% in high-cardiovascular-risk populations, and this effect was independent of administration route (oral/subcutaneous/injection), presence of underlying diabetes, and BMI (125, 127). Furthermore, specifically, a meta-analysis based on US cohort data showed that GLP-1RAs decreased the risk of AF recurrence at 12 months in AF patients who underwent catheter ablation (128). Likewise, another GLP-1RA (albiglutide) has been shown to be associated with AF (129, 130). Finally, a network meta-analysis by Shi et al. that assessed the impact of glucose-lowering agents on AF risk also found that GLP-1RAs significantly reduced the incidence of AF in diabetic patients compared with other glucose-lowering drugs (131).

However, despite the aforementioned studies confirming an association between GLP-1RAs and a reduced risk of AF, consensus remains elusive in current clinical research regarding whether GLP-1RAs exert antiarrhythmic effects, with some studies reporting opposing findings or no significant association. For example, one study found that, among diabetic participants, GLP-1RA use was independently associated with a higher risk of AF events (HR 2.27; 95% CI 1.49–3.47) (132). Similarly, a *post hoc* analysis of the HARMONY trial also demonstrated that albiglutide was associated with an elevated risk of AF and tachyarrhythmias (130). Notably, a meta-analysis of multiple cardiovascular outcome trials (CVOTs) reported no significant difference in AF risk between GLP-1RAs and placebo in participants with type 2 diabetes (OR 0.93; 95% CI 0.70–1.23; *I*^2^ = 58%) (133). Furthermore, while some studies failed to identify a significant correlation between GLP-1RA therapy and overall AF, interestingly, they noted that oral semaglutide exerted a potential protective effect against AF, whereas dulaglutide showed an opposing trend (albeit non-robust), with other GLP-1RAs displaying no significant association with AF (134). In contrast, research by Krychtiuk et al. indicated that, in patients with T2DM, albiglutide did not increase adverse AF events and was instead associated with a trend toward reduced AF incidence during follow-up, although this relationship did not achieve statistical significance (135). Focusing on specific agents, studies also revealed that a *post hoc* analysis of REWIND trial data showed no association between dulaglutide treatment and a reduced incidence of atrial arrhythmias (AAs) in high-risk T2DM populations (136). In addition, Lee et al. reported a case of a female patient with a history of T2DM and paroxysmal AF whose AF exacerbated following a single 0.75 mg dose of dulaglutide (110). Moreover, a study by Satti et al. utilizing data from the TriNetX research database over 12 months reported that preoperative use of GLP-1RAs was not associated with a reduced risk of AF recurrence or ablation-related adverse outcomes (137); however, some scholars have highlighted limitations in this study and have therefore emphasized the need for further research to clarify whether these agents can improve outcomes in AF patients (138) (Tables 1, 2).

**Table 1:** Study on the association between different GLP-1 receptor agonists and atrial fibrillation (human studies).

Author	Research project	Study design	Number of participants	Country of study	Outcome measures assessed	Key study findings
Krychtiuk et al. (135)	Related to albiglutide	Multicenter, double-blind, placebo-controlled trial	9,463	Multiple countries	Primary composite endpoint: cardiovascular death, non-fatal myocardial infarction, non-fatal stroke Secondary endpoints: expanded composite endpoint, heart failure hospitalization, all-cause mortality AF-related endpoints: new-onset AF/atrial flutter events, severe AF events	Albiglutide reduces the risk of major adverse cardiovascular events, independent of baseline AF history (with AF history: aHR = 0.83; without AF history: aHR = 0.77) AF events showed a numerical decreasing trend (HR = 0.82, *P* = 0.12), not reaching statistical significance Does not increase the risk of AF adverse events
Wu et al. (139)	Study on the clinical effectiveness of tirzepatide	Retrospective cohort study; propensity score matching; Cox proportional hazards model	5,597 matched pairs	Based on the TriNetX Global Collaborative Network	Primary composite endpoint: cardioversion, intravenous antiarrhythmic drug use, AF ablation Secondary endpoints: heart failure, ischemic stroke, all-cause mortality Subgroup analysis	Tirzepatide significantly reduces the risk of the primary composite endpoint (HR = 0.65, *P* < 0.001) Significantly reduces the risks of heart failure (HR = 0.60) and all-cause mortality (HR = 0.35) Consistent effects in patients with different AF subtypes, comorbid coronary heart disease/obesity/chronic kidney disease
Glaser et al. (140)	Systematic review on the impact of GLP-1 RAs on AF incidence	Systematic review	Involving semaglutide (90,299), liraglutide (84,532), dulaglutide (83,894)	Based on published global studies	Impact of each GLP-1 RA (semaglutide, liraglutide, dulaglutide) on AF incidence Comparative effectiveness with SGLT2 inhibitors	Semaglutide consistently reduces AF incidence (maximum reduction of 42%) Liraglutide shows inconsistent effects, with some studies suggesting increased AF risk Dulaglutide has no significant AF-reducing effect, and some show an increasing trend SGLT2 inhibitors have a more stable effect in reducing AF/atrial flutter
Cesaro et al. (127)	Systematic review and meta-analysis on the impact of semaglutide on new-onset atrial fibrillation (AF)	Systematic review and meta-analysis	26 RCTs, totaling 48,583 participants (25,879 in the semaglutide group, 22,704 in the control group)	Based on global RCT data	Primary outcome: new-onset AF; subgroup analysis (formulation type, administration route, concurrent SGLT2i treatment, etc.)	Semaglutide significantly reduces the risk of new-onset AF (OR=0.83, *P* = 0.03) with no heterogeneity (*I*^2^ = 0%) Oral formulations show more significant effects (OR=0.48, *P* = 0.04), and the active control group shows a long-term reduction of 59% (OR = 0.41, *P* = 0.01) When not combined with SGLT2i treatment, AF risk is reduced by 21% (OR = 0.79, *P* = 0.04) Baseline BMI and HbA1c do not affect treatment effects
Guo et al. (141)	Study on the impact of semaglutide on the recurrence of atrial arrhythmias after AF ablation	Prospective study; patients divided into two groups based on preference; 12-month follow-up (including 3-month blanking period)	437 (158 in the semaglutide group, 279 in the control group)	China	Primary outcome: recurrence rate of atrial arrhythmias within 12 months Secondary outcomes: AF recurrence rate, weight change, glycated hemoglobin change, New York Heart Association functional class change Safety indicators: procedure-related adverse events, semaglutide-related adverse events	The semaglutide group has a higher non-recurrence rate of atrial arrhythmias (HR = 0.68, *P* = 0.030) The semaglutide group shows more significant weight reduction (−8.2% vs. −4.6%) and glycated hemoglobin reduction (−1.3% vs. −0.6%) (both *P* < 0.001) 43.83% of the semaglutide group experience gastrointestinal symptoms, and 11.6% discontinue treatment due to adverse events
Hamedi et al. (133)	Study on GLP-1 receptor agonists and atrial fibrillation in cardiovascular outcome trials	Meta-analysis; random-effects model; including CVOTs studies	41,206 (20,598 in the GLP-1 RA group, 20,608 in the placebo group)	Based on multinational CVOTs data	Primary outcome: incidence of new-onset atrial fibrillation among serious adverse events	The incidence of new-onset AF in the GLP-1 RA group is 1.35%, and 1.37% in the placebo group Pooled analysis shows no significant difference in AF incidence between GLP-1 RA and placebo (RR = 0.93, 95% CI 0.70–1.23, *P* = 0.60) In subgroups, the REWIND trial shows an increased AF risk in the dulaglutide group (RR = 1.45), with no significant differences in other trials
Raubenheimer et al. (136)	Impact of dulaglutide on new-onset atrial fibrillation or atrial flutter in patients with type 2 diabetes (post hoc analysis of the REWIND trial)	Multicenter, double-blinded, placebo-controlled randomized trial; *post hoc* analysis; intention-to-treat population	9,543 (4,769 in the dulaglutide group, 4,774 in the placebo group)	Twenty-four countries	Primary outcome: new-onset atrial fibrillation/atrial flutter (confirmed by annual electrocardiogram) Composite outcome: atrial arrhythmia combined with death, cardiovascular death, stroke, heart failure	No significant difference in the incidence of new-onset atrial arrhythmias between the dulaglutide group and the placebo group (5.6% vs. 5.3%, HR = 1.05, *P* = 0.59) In subgroup analysis, the risk of atrial arrhythmias is slightly increased in drinkers and *β*-blocker users, but the difference is not statistically significant No significant between-group difference in composite outcomes
Monami et al. (129)	Systematic review and meta-analysis of GLP-1 receptor agonists and atrial fibrillation	Systematic review and meta-analysis, including randomized controlled trials with duration ≥12 weeks; random-effects model	31 valid trials, totaling 33,271 participants (17,966 in the GLP-1 RA group, 15,305 in the control group)	Based on global randomized controlled trial data	Primary outcome: incidence of atrial fibrillation (based on serious adverse events or confirmed cases)	GLP-1 receptor agonists as a class do not increase the risk of atrial fibrillation (OR = 0.87, *P* = 0.15) In subgroup analysis, albiglutide shows a trend of increased risk, but does not reach statistical significance Other drugs (exenatide, liraglutide, dulaglutide, etc.) have no significant impact
Venier et al. (142)	Impact of GLP-1 receptor agonist therapy on atrial fibrillation recurrence after catheter ablation in obese patients (real-world data analysis)	Retrospective cohort study; TriNetX database; 1:1 propensity score matching (incorporating 82 clinical and demographic variables)	6,700 participants (3,350 in the GLP-1 RA group and 3,350 in the non-use group after matching)	Based on the TriNetX Research Network	Primary outcome: Atrial fibrillation recurrence (confirmed by hospitalization or outpatient visit) Secondary outcomes: Progression to permanent atrial fibrillation, all-cause mortality, heart failure hospitalization, cardiovascular-related hospitalization, repeat ablation, etc.	The GLP-1 RA group had a lower atrial fibrillation recurrence rate (6.66% vs. 7.72%, HR = 0.82, *P* < 0.0001) The risks of progression to permanent atrial fibrillation, all-cause mortality, heart failure hospitalization, and cardiovascular-related hospitalization were all significantly reduced (all *P* < 0.01) No significant difference was observed in the repeat ablation rate (*P* = 0.15)
Saglietto et al. (125)	Systematic review and meta-analysis on semaglutide (GLP-1 receptor agonist) reducing AF incidence	Systematic review and meta-analysis, including randomized controlled trials; random-effects model	12,651 (7,285 in the semaglutide group, 5,366 in the placebo group)	Based on global randomized controlled trial data	Primary outcome: incidence of new-onset atrial fibrillation Secondary outcome: incidence of ischemic stroke Subgroup analysis (administration route: oral vs. subcutaneous injection)	Semaglutide reduces the risk of new-onset AF by 42% (RR = 0.58, 95% CI 0.40–0.85, *I*^2^ = 0%) No significant difference in efficacy between oral and subcutaneous administration (*P* = 0.83) Baseline diabetes status and BMI do not affect treatment effects4. No significant between-group difference in the incidence of ischemic stroke
Satti et al. (137)	Impact of GLP-1 receptor agonists on AF recurrence after catheter ablation	Retrospective cohort study; TriNetX database; 1:1 propensity score matching	3,250 (1,625 in the GLP-1 RA group, 1,625 in the non-use group after matching)	United States (based on the TriNetX research network, mainly US medical organizations)	Primary composite outcome: cardioversion, new initiation of class I/III antiarrhythmic drug therapy, or re-ablation after a 3-month blanking period Secondary outcomes: ischemic stroke, all-cause hospitalization, all-cause mortality	No significant difference in the risk of the primary composite outcome between the GLP-1 RA group and the non-use group (HR = 1.04, 95% CI 0.92–1.19, *P* = 0.51) No significant between-group differences in secondary outcomes such as ischemic stroke, all-cause hospitalization, and all-cause mortality
Wu et al. (134)	Association between GLP-1 receptor agonists and cardiac arrhythmias in patients with type 2 diabetes or obesity (systematic review and meta-analysis)	Systematic review and meta-analysis, including randomized controlled trials; fixed-effects model	79,720 (44,028 in the GLP-1 RA group, 35,692 in the control group)	Based on global randomized controlled trial data	Primary outcomes: new-onset atrial fibrillation (AF), atrial flutter (AFL), ventricular arrhythmias (VAs), sudden cardiac death (SCD) Subgroup analysis (drug type, dosage, baseline BMI, etc.)	GLP-1 RAs as a class do not increase the risks of AF, AFL, VAs, or SCD (RR = 0.97, 0.83, 1.24, 0.89 ,respectively, all *P*’s > 0.05) In subgroups, dulaglutide shows a trend of increased AF (RR = 1.40, *P* = 0.03), and oral semaglutide shows a trend of reduced AF (RR = 0.43, *P* = 0.02) High-dose GLP-1 RAs (RR = 1.63, *P* = 0.01) and high baseline BMI (RR = 1.60, *P* = 0.03) significantly increase the risk of VAs
Zhang et al. (124)	Association between GLP-1 receptor agonists and atrial fibrillation, cardiac arrest, and ventricular fibrillation (causal evidence from drug target Mendelian randomization)	Two-sample Mendelian randomization analysis; genetic instrumental variables derived from cis-expression quantitative trait loci of the *GLP1R* gene; supplementary Bayesian colocalization and multivariable Mendelian randomization analysis	Genetic instrumental variables based on 31,684 European populations; outcome data from FinnGen (AF: 50,743 cases/210,652 controls) and GWAS Catalog (cardiac arrest/ventricular fibrillation: 1,137 cases/380,919 controls)	Based on European population genetic data and global GWAS data	Primary outcome: atrial fibrillation (AF) Secondary outcomes: cardiac arrest, ventricular fibrillation	Genetically proxied GLP-1 RAs are associated with a reduced risk of AF (OR=0.78, *P* = 4.45 ×10^−8^) Associated with a reduced risk of cardiac arrest and ventricular fibrillation (OR = 0.60, *P* = 0.0039) Bayesian colocalization analysis shows no shared genetic variants, and the association is not statistically significant after adjusting for BMI and type 2 diabetes

**Table 2:** Study on the association between different GLP-1 receptor agonists and atrial fibrillation (animal experiments).

Author	Research project	Study design	Number of participants	Country of study	Outcome measures assessed	Key study findings
Chan et al. (143)	Study on the impact of GLP-1 receptor agonists on pulmonary vein arrhythmogenesis and calcium homeostasis	Animal experiment (rabbit pulmonary vein tissue and cardiomyocytes); conventional microelectrode technique, whole-cell patch-clamp technique	Rabbit pulmonary vein tissue (*n* = 8/group), rabbit pulmonary vein cardiomyocytes (*n* = 9–14/index)	Taiwan, China	Pulmonary vein spontaneous activity, sinoatrial node electrical activity Ion currents (L-type calcium current, sodium–calcium exchanger current, sodium current, late sodium current) Sarcoplasmic reticulum calcium content Antiarrhythmic effects after inhibitor intervention	GLP-1 receptor agonist concentration-dependently reduces pulmonary vein spontaneous activity without affecting sinoatrial node function Reduces L-type calcium current, sodium–calcium exchanger current, late sodium current, and increases sarcoplasmic reticulum calcium content PKA, CaMKII, and NCX inhibitors can weaken its antiarrhythmic effects
Nakamura et al. (122)	Study on the inhibitory effect of liraglutide on atrial electrophysiological changes	Animal experiment (beagle dog AF model); 3-week rapid atrial pacing; divided into the liraglutide group and pacing control group	Seven beagle dogs (four in the liraglutide group, three in the pacing control group)	Japan	Primary outcomes: atrial effective refractory period (AERP), conduction velocity (CV), AF inducibility Hemodynamic indicators (blood pressure, cardiac output, etc.)	The AF inducibility in the liraglutide group is significantly lower than that in the control group (5 ± 9% vs. 73 ± 5%, *P* = 0.0262) The conduction velocity in the liraglutide group is significantly higher than that in the control group (at 2 and 3 weeks, both *P* < 0.05) No significant difference in the degree of AERP shortening between the two groups (*P* = 0.5926)
Wang et al. (121)	Liraglutide inhibits AngII-induced cardiac fibroblast proliferation and extracellular matrix (ECM) deposition by regulating the miR-21/PTEN/PI3K pathway	Cell experiment (mouse atrial fibroblasts); divided into control group, AngII group, AngII + different concentrations of liraglutide groups, AngII + liraglutide + miR-21 mimic group, etc.	Mouse atrial fibroblasts (cultured *in vitro*)	China	Primary outcomes: cardiac fibroblast proliferation, migration, and invasion abilities ECM deposition-related indicators (mRNA and protein expressions of COL-I, COL-III, *α*-SMA, TGF-β1) Expressions of molecules related to the miR-21/PTEN/PI3K pathway	Liraglutide concentration-dependently inhibits AngII-induced fibroblast proliferation, migration, and invasion Liraglutide reduces AngII-induced miR-21 expression, increases PTEN expression, and inhibits PI3K/AKT pathway activation Overexpression of miR-21 can reverse the above protective effects of liraglutide
Zhou et al. (144)	Exenatide reduces AF susceptibility by inhibiting hKv1.5 and hNav1.5 channels	Cell experiments (HEK293 cells, rat atrial cardiomyocytes) + animal experiments (rat AF model); using whole-cell patch-clamp, electrophysiological mapping, and other techniques	Cell experiments: HEK293 cells (stably expressing hKv1.5 or hNav1.5), rat atrial cardiomyocytes Animal experiments: SD rats (♂, 220 ± 10 g)	China	Primary outcomes: hKv1.5 and hNav1.5 channel currents, action potential duration (APD), AF incidence and duration Secondary outcomes: other myocardial ion channel currents (I_Ca,L, I_to, I_ss, etc.)	Exenatide reversibly inhibits hKv1.5 (IC₅₀ = 3.08 μM) and hNav1.5 (IC₅₀ = 3.30 μM) channel currents; Prolongs the action potential duration of rat atrial cardiomyocytes and inhibits I_ss and I_to currents Significantly reduces the incidence and duration of AF in rats and improves atrial conduction disorders
Bohne et al. (29)	Protective effect of glucagon-like peptide-1 (GLP-1) and liraglutide on AF and atrial remodeling in type 2 diabetic mice	Animal experiment (type 2 diabetic db/db mouse model); divided into wild-type group, db/db saline group, db/db GLP-1 treatment group, db/db liraglutide treatment group; chronic administration (4 weeks)	GLP-1 treatment experiment: wild-type mice (*n* = 25), db/db saline group (*n* = 26), db/db GLP-1 group (*n* = 28) Liraglutide treatment experiment: wild-type mice (*n* = 13), db/db vehicle group (*n* = 16), db/db liraglutide group (*n* = 11)	Canada	Primary outcomes: AF inducibility, duration, atrial effective refractory period (AERP) Electrophysiological indicators (P-wave duration, atrial conduction velocity, action potential duration) Structural remodeling indicators (atrial fibrosis degree, collagen gene expression)	Both GLP-1 and liraglutide reduce AF inducibility and duration in db/db mice, shorten P-wave duration, and improve atrial conduction velocity Reverse the prolongation of atrial action potential duration in db/db mice and restore repolarizing K^+^ currents (I_to, I_Kur); Significantly reduce atrial fibrosis and decrease *Col1a* and *Col3a* gene expressions

Regarding the inconsistency of the aforementioned results, we hypothesize that it may be attributed to the following factors: (1) Drug structure and receptor binding specificity: Inherent variations exist in the molecular structure, half-life, and receptor binding efficacy among different GLP-1RAs, which directly determine their functional selectivity for atrial cardiomyocytes. (2) Variations in myocardial metabolic regulatory capacity: GLP-1RAs exhibit heterogeneity in their regulation of myocardial fatty acid oxidation, glucose metabolism, and mitochondrial function. (3) Weight loss effect and dissociation of independent effects: GLP-1RAs generally possess weight-loss properties, and obesity is a key risk factor for AF; however, the AF-related effects of certain agents are independent of weight loss. (4) Heterogeneity in autonomic nervous system modulation: Different formulations vary in the extent to which they interfere with sympathetic nerve activity and the cardiac sympathetic–vagal balance. (5) Heterogeneity in the regulation of EAT inflammation: EAT inflammation is a crucial predisposing factor for AF, and GLP-1RAs differ in their capacity to regulate EAT inflammation. (6) Differences in study design, statistical methodologies, and population characteristics: Even for the same drug, disparate conclusions may be derived from different studies (17, 116, 130, 133, 134, 145).

### 5.3 Mechanisms underlying the association between GLP-1RAs and AF

A study has demonstrated that patients receiving GLP-1RA therapy exhibit a sustained increase in heart rate (146). A study by Lubberding et al. further confirmed that GLP-1 exerts a positive chronotropic effect by directly activating GLP-1 receptors expressed in sinoatrial node pacemaker cells. This mechanism is independent of both the autonomic nervous system and hyperpolarization-activated cyclic nucleotide-gated channels. Instead, GLP-1 modulates calcium signaling pathways through protein kinase A (PKA)-dependent phosphorylation of calcium-cycling proteins, thereby altering action potential morphology and dominant pacemaker sites, ultimately leading to an increase in heart rate (147). Despite the observed increase in heart rate, several meta-analyses have demonstrated that GLP-1RAs are associated with a lower risk of AF compared with other glucose-lowering agents, suggesting their potential protective effect against AF occurrence (131, 148). Nevertheless, the direct role of GLP-1RAs in AF pathogenesis remains elusive. Building on previous research, we hypothesize that their effects on AF may be attributed to the following mechanisms.

#### 5.3.1 Regulation of electrophysiological properties and atrial remodeling

GLP-1RAs can shorten the effective refractory period, reduce conduction velocity, and diminish AF inducibility (122) while counteracting the effects of heart failure on APD, AF inducibility, and structural remodeling—specifically, by reducing left atrial volume and alleviating fibrosis (149). In addition, GLP-1RAs are closely associated with atrial remodeling, including mitigating EAT accumulation, suppressing inflammatory responses, lowering blood concentrations of AGEs and fibrotic factors, and regulating serum calcium levels (150, 151), thereby collectively inhibiting AF progression from both electrophysiological and structural aspects.

#### 5.3.2 Regulation of calcium homeostasis and its role in inhibiting pulmonary vein automaticity

GLP-1RAs mitigate high glucose-induced cardiomyocyte apoptosis by enhancing sarcoplasmic reticulum/endoplasmic reticulum calcium ATPase function (152). They also significantly reduce phosphorylation of the ryanodine receptor (RyR) at the S2814 site and lower the risk of triggered activity and atrial arrhythmias by suppressing calcium leakage (153), thereby reflecting their antiarrhythmic properties. These calcium-regulatory effects also contribute to inhibiting pulmonary vein (PV) automaticity: as a primary trigger of atrial fibrillation, PVs are modulated by GLP-1RAs, which affect PV electrical activity, ionic properties, and intracellular calcium homeostasis to suppress PV automaticity (143). Given that abnormal automaticity and micro-reentry induction are key characteristics of AF initiation and maintenance (154, 155), it is hypothesized that GLP-1RAs may reduce AF risk by decreasing PV automaticity. Calcium ions play a pivotal role in this process, with cyclic adenosine monophosphate-dependent PKA, CaMKII pathways, and NCX—activated via *β*1-adrenergic receptor stimulation—mediating this mechanism (143).

#### 5.3.3 Improvement of myocardial metabolism, associated protective effects, and indirect impact on CAD

GLP-1RAs enhance mitochondrial function in cardiomyocytes, mitigate oxidative stress and cellular damage, improve myocardial fatty acid metabolism, stabilize blood glucose levels, and regulate myocardial mitochondrial function via the renin–angiotensin–aldosterone system (156, 157). Collectively, these effects optimize myocardial energy metabolism to reduce AF triggers. Furthermore, their combined glucose-lowering and weight-reduction effects reduce the risk of AF onset and recurrence and further contribute to the regulation of AF triggers through metabolic modulation. GLP-1RAs exert a protective effect on CAD. Given that CAD increases atrial tissue excitability by promoting reentry, exacerbating ischemia, and inducing electrical heterogeneity—thus accelerating AF progression—their protective action against CAD may indirectly lower AF risk (158, 159).

## 6 Role of GLP-1RAs in EAT and underlying mechanisms

A study has demonstrated that GLP-1R is highly expressed in the cardiac atria (160) and is concurrently expressed in human EAT. RNA sequencing analyses have confirmed GLP-1R gene expression in EAT, and immunofluorescence studies have further validated the presence of GLP-1R protein, whereas no such signal has been detected in subcutaneous fat (95). In addition, Dozio et al. conducted RNA sequencing on EAT samples obtained during cardiac surgery and similarly identified GLP-1 receptor expression in human EAT (161), providing a molecular basis for the action of GLP-1RAs on EAT. Moreover, GLP-1R in EAT positively correlates with genes associated with brown adipose tissue activity, FA oxidation, and white-to-brown adipocyte differentiation, while showing a negative correlation with pro-adipogenic genes (95, 161). These findings suggest that GLP-1RAs may target GLP-1R in EAT to regulate local adipogenesis, energy utilization, and cellular phenotype, thereby influencing cardiovascular health (Figure 2).

Multiple studies have demonstrated that GLP-1RAs significantly reduce the thickness or volume of EAT: once-weekly GLP-1RAs (semaglutide, dulaglutide) significantly decrease ultrasound-measured EAT thickness in a dose-dependent manner (162). Treatment with semaglutide, dulaglutide, or liraglutide has been shown to reduce EAT volume in obese patients with T2DM, with the magnitude of EAT reduction exceeding that attributable to overall weight loss (26, 162). Notably, the addition of liraglutide to metformin reduces EAT by nearly 40%, with approximately 30% of this reduction achieved as early as 3 months after treatment initiation (26). Similarly, liraglutide monotherapy for 3 months significantly reduced EAT thickness in obese T2DM patients, and this reduction was positively correlated with decreases in BMI (163). In addition, studies have reported that first-generation once-weekly GLP-1RAs (e.g., exenatide) reduce EAT and hepatic triglyceride content (164). Meta-analyses further corroborate this effect: compared with SGLT2 inhibitors or statins, GLP-1RAs are associated with more pronounced changes in EAT, with stronger beneficial effects following long-term (over 6 months) administration. In younger patients, EAT reduction is more significant with increasing BMI (165).

Mechanistically, GLP-1RAs may promote the differentiation of EAT preadipocytes, improve insulin sensitivity, stimulate EAT thermogenesis and adipocyte browning, and enhance fatty acid oxidation (162). Brown adipose tissue plays a protective role under stress conditions such as ischemia and hypoxia (166). Consequently, GLP-1RAs may regulate EAT via multiple pathways, including suppression of local adipogenesis, increased fat utilization, and induction of white-to-brown adipocyte differentiation (95). This regulation is likely associated with cardiovascular protective effects; for instance, reducing inflammation in EAT surrounding the left atrium and coronary arteries and enhancing fatty acid oxidation may help prevent atrial fibrillation and coronary artery disease (63). Furthermore, studies have demonstrated that the therapeutic effects of GLP-1RAs and GLP-1/GLP-2 receptor dual agonists against myocardial ischemia/reperfusion injury are, at least partially, dependent on EAT regulation (99). Based on these findings, it is hypothesized that certain effects of GLP-1RAs are adipose tissue-specific and may target EAT. Given that GLP-1Rs are expressed in adipose tissue—with elevated mRNA and protein expression levels in visceral adipose tissue (167, 168), their actions may exhibit visceral adipose tissue specificity.

However, despite our partial elaboration on the relationship between GLP-1RAs and EAT, several studies have demonstrated that liraglutide exerts no significant effect on EAT. For example, following 26 weeks of treatment in South Asian patients with T2DM, liraglutide reduced visceral fat and improved glycemic control yet did not exert a notable impact on EAT volume (169). Similarly, a subgroup analysis of the MAGNA VICTORIA study revealed that T2DM patients treated with liraglutide for 26 weeks experienced reductions in body weight and subcutaneous fat, yet showed no change in EAT accumulation (170). In addition, a meta-analysis encompassing 16 randomized clinical trials indicated that while liraglutide reduced visceral and hepatic fat volume, its effect of EAT reduction did not reach statistical significance (171).

Regarding the aforementioned inconsistent findings across different studies on liraglutide, a study proposes that this may be attributed to factors including population specificity, variations in study design, and discrepancies in intervention and measurement conditions (169, 170). In conclusion, the specific mechanism by which GLP-1RAs act on EAT remains elusive. Current evidence suggests that modulation of EAT may serve as a key mediating pathway for the cardioprotective effects of GLP-1RAs, and this area warrants further investigation.

## 7 Future perspectives

Despite the promising potential of GLP-1RAs for the treatment of AF by targeting EAT, numerous significant challenges remain for their clinical translation. First, comparative studies investigating the effects of different GLP-1RA agents on EAT are relatively scarce. The specific differences in their efficacy in reducing EAT volume, improving EAT inflammatory status, and ultimately preventing and treating AF remain unclear—a gap that greatly limits clinicians' ability to individualize drug selection based on patient-specific conditions. Second, significant heterogeneity in therapeutic responses to GLP-1RAs across individuals has been observed. Particularly in patients of different ethnicities or with distinct metabolic backgrounds, the effects of GLP-1RAs on EAT and their impact on AF risk vary substantially, thereby making it difficult to develop a unified and effective treatment regimen. Third, the specific molecular mechanisms underlying GLP-1RA-mediated regulation of EAT have not been fully elucidated. For instance, it remains to be investigated whether GLP-1RAs act solely through direct binding to GLP-1 receptors on EAT cells or indirectly modulate EAT by regulating systemic metabolic status, as well as which precise transduction mechanisms of intracellular signaling pathways mediate this process. In addition, existing imaging techniques for EAT assessment lack unified standards and specifications regarding the accuracy of EAT volume measurement, the precision of density analysis, and the comprehensiveness of functional evaluation. This limitation partially compromises the comparability of study results and the reliability of clinical applications. Finally, the clinical role of GLP-1RAs in AF treatment has not been clearly defined. For example, critical issues such as optimal timing, dose adjustment, and drug–drug interactions when combining GLP-1RAs with traditional AF treatments or other cardiovascular drugs require further large-scale clinical studies to be established.

Addressing these issues is crucial to advancing the effective management of AF via GLP-1RA-mediated EAT targeting, which will provide a more solid theoretical and practical foundation for clinical practice.

## 8 Conclusion

In conclusion, as a key metabolic target of AF, abnormal activation of EAT plays a central role in the vicious cycle linking metabolic disorders and cardiac arrhythmias. GLP-1RAs hold potential value for AF prevention and treatment by regulating EAT volume and metabolic phenotype and by improving the local inflammatory microenvironment, as supported by partial evidence from basic and clinical studies. However, the clinical translation of GLP-1RAs remains constrained by several challenges, such as unclear drug-specific differences, significant heterogeneity in individual therapeutic responses, incompletely elucidated molecular mechanisms, the absence of unified standards for EAT assessment, and ambiguous clinical positioning. In the future, targeted research is needed to address these issues, further consolidate the theoretical and practical basis of GLP-1RAs targeting EAT for AF treatment, and provide new strategies for the clinical management of AF patients, especially those with comorbid metabolic diseases.

## References

[1] Tsao CW Aday AW Almarzooq ZI Anderson CAM Arora P Avery CL Heart disease and stroke statistics – 2023 update: a report from the American Heart Association. Circulation. (2023) 147(8):e93–621. 10.1161/CIR.0000000000001123 36695182 PMC12135016

[2] Writing Committee Members, Joglar JA Chung MK Armbruster AL Benjamin EJ Chyou JY Cronin EM 2023 ACC/AHA/ACCP/HRS guideline for the diagnosis and management of atrial fibrillation: a report of the American College of Cardiology/American Heart Association joint committee on clinical practice guidelines. J Am Coll Cardiol. (2024) 83(1):109–279. 10.1016/j.jacc.2023.08.017 38043043 PMC11104284

[3] CDC. National Diabetes Statistics Report. Diabetes. (2024). Available online at: https://www.cdc.gov/diabetes/php/data-research/index.html (Accessed August 11, 2025).

[4] Aune D Feng T Schlesinger S Janszky I Norat T Riboli E. Diabetes mellitus, blood glucose and the risk of atrial fibrillation: a systematic review and meta-analysis of cohort studies. J Diabetes Complications. (2018) 32(5):501–11. 10.1016/j.jdiacomp.2018.02.004 29653902

[5] Hindricks G Potpara T Dagres N Arbelo E Bax JJ Blomström-Lundqvist C Corrigendum to: 2020 ESC guidelines for the diagnosis and management of atrial fibrillation developed in collaboration with the European Association for Cardio-Thoracic Surgery (EACTS): the Task Force for the diagnosis and management of atrial fibrillation of the European Society of Cardiology (ESC) developed with the special contribution of the European Heart Rhythm Association (EHRA) of the ESC. Eur Heart J. (2021) 42(40):4194. 10.1093/eurheartj/ehab648 34520521

[6] Kirchhof P Benussi S Kotecha D Heidbuchel H. 2016 ESC guidelines for the management of atrial fibrillation developed in collaboration with EACTS. Rev Esp Cardiol (Engl Ed). (2017) 70(1):50. 10.1016/j.rec.2016.11.033 28038729

[7] Movahed MR Hashemzadeh M Jamal MM. Diabetes mellitus is a strong, independent risk for atrial fibrillation and flutter in addition to other cardiovascular disease. Int J Cardiol. (2005) 105(3):315–8. 10.1016/j.ijcard.2005.02.050 16274775

[8] Okunrintemi V Mishriky BM Powell JR Cummings DM. Sodium-glucose co-transporter-2 inhibitors and atrial fibrillation in the cardiovascular and renal outcome trials. Diabetes Obes Metab. (2021) 23(1):276–80. 10.1111/dom.14211 33001548

[9] Seyed Ahmadi S Svensson AM Pivodic A Rosengren A Lind M. Risk of atrial fibrillation in persons with type 2 diabetes and the excess risk in relation to glycaemic control and renal function: a Swedish cohort study. Cardiovasc Diabetol. (2020) 19(1):9. 10.1186/s12933-019-0983-1 31954408 PMC6969407

[10] Dahlqvist S Rosengren A Gudbjörnsdottir S Pivodic A Wedel H Kosiborod M Risk of atrial fibrillation in people with type 1 diabetes compared with matched controls from the general population: a prospective case-control study. Lancet Diabetes Endocrinol. (2017) 5(10):799–807. 10.1016/S2213-8587(17)30262-0 28838683

[11] Bisson A Bodin A Fauchier G Herbert J Angoulvant D Ducluzeau PH Sex, age, type of diabetes and incidence of atrial fibrillation in patients with diabetes mellitus: a nationwide analysis. Cardiovasc Diabetol. (2021) 20(1):24. 10.1186/s12933-021-01216-7 33482830 PMC7821402

[12] Obesity and overweight. Available online at: https://www.who.int/news-room/fact-sheets/detail/obesity-and-overweight (Accessed August 9, 2025).

[13] Nyström PK Carlsson AC Leander K de Faire U Hellenius ML Gigante B. Obesity, metabolic syndrome and risk of atrial fibrillation: a Swedish, prospective cohort study. PLoS One. (2015) 10(5):e0127111. 10.1371/journal.pone.0127111 25978738 PMC4433194

[14] Lee H Choi EK Lee SH Han K-D Rhee T-M Park C-S Atrial fibrillation risk in metabolically healthy obesity: a nationwide population-based study. Int J Cardiol. (2017) 240:221–7. 10.1016/j.ijcard.2017.03.103 28385358

[15] Feng T Vegard M Strand LB Laugsand LE Mørkedal B Aune D Metabolically healthy obesity and risk for atrial fibrillation: the HUNT study. Obesity (Silver Spring). (2019) 27(2):332–8. 10.1002/oby.22377 30605242

[16] McAninch EA Fonseca TL Poggioli R Panos AL Salerno TA Deng Y Epicardial adipose tissue has a unique transcriptome modified in severe coronary artery disease. Obesity (Silver Spring). (2015) 23(6):1267–78. 10.1002/oby.21059 25959145 PMC5003780

[17] Iacobellis G. Epicardial fat inflammation and GLP-1/GIP receptor analogs: are we shifting our perspective? Curr Cardiol Rep. (2025) 27(1):161. 10.1007/s11886-025-02325-5 41307857

[18] Packer M. Epicardial adipose tissue may mediate deleterious effects of obesity and inflammation on the myocardium. J Am Coll Cardiol. (2018) 71(20):2360–72. 10.1016/j.jacc.2018.03.509 29773163

[19] Mazurek T Zhang L Zalewski A Mannion JD Diehl JT Arafat H Human epicardial adipose tissue is a source of inflammatory mediators. Circulation. (2003) 108(20):2460–6. 10.1161/01.CIR.0000099542.57313.C5 14581396

[20] Nalliah CJ Bell JR Raaijmakers AJA Waddell HM Wells SP Bernasochi GB Epicardial adipose tissue accumulation confers atrial conduction abnormality. J Am Coll Cardiol. (2020) 76(10):1197–211. 10.1016/j.jacc.2020.07.017 32883413

[21] Venteclef N Guglielmi V Balse E Gaborit B Cotillard A Atassi F Human epicardial adipose tissue induces fibrosis of the atrial myocardium through the secretion of adipo-fibrokines. Eur Heart J. (2015) 36(13):795–805. 10.1093/eurheartj/eht099 23525094

[22] Iacobellis G. Epicardial fat: a new cardiovascular therapeutic target. Curr Opin Pharmacol. (2016) 27:13–8. 10.1016/j.coph.2016.01.004 26848943

[23] Kristensen SL Rørth R Jhund PS Docherty KF Sattar N Preiss D Cardiovascular, mortality, and kidney outcomes with GLP-1 receptor agonists in patients with type 2 diabetes: a systematic review and meta-analysis of cardiovascular outcome trials. Lancet Diabetes Endocrinol. (2019) 7(10):776–85. 10.1016/S2213-8587(19)30249-9 31422062

[24] Gerstein HC Colhoun HM Dagenais GR Diaz R Lakshmanan M Pais P Dulaglutide and cardiovascular outcomes in type 2 diabetes (REWIND): a double-blind, randomised placebo-controlled trial. Lancet. (2019) 394(10193):121–30. 10.1016/S0140-6736(19)31149-3 31189511

[25] Morano S Romagnoli E Filardi T Nieddu L Mandosi E Fallarino M Short-term effects of glucagon-like peptide 1 (GLP-1) receptor agonists on fat distribution in patients with type 2 diabetes mellitus: an ultrasonography study. Acta Diabetol. (2015) 52(4):727–32. 10.1007/s00592-014-0710-z 25577244

[26] Iacobellis G Mohseni M Bianco SD Banga PK. Liraglutide causes large and rapid epicardial fat reduction. Obesity (Silver Spring). (2017) 25(2):311–6. 10.1002/oby.21718 28124506

[27] Malavazos AE Iacobellis G Dozio E Basilico S Di Vincenzo A Dubini C Human epicardial adipose tissue expresses glucose-dependent insulinotropic polypeptide, glucagon, and glucagon-like peptide-1 receptors as potential targets of pleiotropic therapies. Eur J Prev Cardiol. (2023) 30(8):680–93. 10.1093/eurjpc/zwad050 36799940

[28] Iacobellis G Camarena V Sant DW Wang G. Human epicardial fat expresses glucagon-like peptide 1 and 2 receptors genes. Horm Metab Res. (2017) 49(8):625–30. 10.1055/s-0043-109563 28514806 PMC7430146

[29] Bohne LJ Jansen HJ Dorey TW Daniel IM Jamieson KL Belke DD Glucagon-like peptide-1 protects against atrial fibrillation and atrial remodeling in type 2 diabetic mice. JACC Basic Transl Sci. (2023) 8(8):922–36. 10.1016/j.jacbts.2023.01.005 37719430 PMC10504404

[30] Hindricks G Potpara T Dagres N Arbelo E Bax JJ Blomström-Lundqvist C 2020 ESC guidelines for the diagnosis and management of atrial fibrillation developed in collaboration with the European Association for Cardio-Thoracic Surgery (EACTS): the task force for the diagnosis and management of atrial fibrillation of the European Society of Cardiology (ESC) developed with the special contribution of the European Heart Rhythm Association (EHRA) of the ESC. Eur Heart J. (2021) 42(5):373–498. 10.1093/eurheartj/ehaa612 32860505

[31] Nielsen JC Johannessen A Raatikainen P Hindricks G Walfridsson H Pehrson SM Long-term efficacy of catheter ablation as first-line therapy for paroxysmal atrial fibrillation: 5-year outcome in a randomised clinical trial. Heart. (2017) 103(5):368–76. 10.1136/heartjnl-2016-309781 27566295

[32] De Maat GE Mulder BA Berretty WL Al-Jazairi MIH Tan YES Wiesfeld ACP Obesity is associated with impaired long-term success of pulmonary vein isolation: a plea for risk factor management before ablation. Open Heart. (2018) 5(1):e000771. 10.1136/openhrt-2017-000771 29862033 PMC5976117

[33] Zhou J Sun L Chen L Liu S Zhong L Cui M. Comprehensive metabolomic and proteomic analyses reveal candidate biomarkers and related metabolic networks in atrial fibrillation. Metabolomics. (2019) 15(7):96. 10.1007/s11306-019-1557-7 31227919

[34] Lenski M Schleider G Kohlhaas M Adrian L Adam O Tian Q Arrhythmia causes lipid accumulation and reduced glucose uptake. Basic Res Cardiol. (2015) 110(4):40. 10.1007/s00395-015-0497-2 26018791

[35] Shingu Y Takada S Yokota T Shirakawa R Yamada A Ooka T Correlation between increased atrial expression of genes related to fatty acid metabolism and autophagy in patients with chronic atrial fibrillation. PLoS One. (2020) 15(4):e0224713. 10.1371/journal.pone.0224713 32315296 PMC7173849

[36] Liu GZ Hou TT Yuan Y Hang P Zhao J Sun L Fenofibrate inhibits atrial metabolic remodelling in atrial fibrillation through PPAR-α/sirtuin 1/PGC-1*α* pathway. Br J Pharmacol. (2016) 173(6):1095–109. 10.1111/bph.13438 26787506 PMC5341245

[37] Li X Yang X Li Y Yuan M Tian C Zhang X Mitochondria and the pathophysiological mechanism of atrial fibrillation. Curr Pharm Des. (2018) 24(26):3055–61. 10.2174/1381612824666180903125300 30179127

[38] Mason FE Pronto JRD Alhussini K Maack C Voigt N. Cellular and mitochondrial mechanisms of atrial fibrillation. Basic Res Cardiol. (2020) 115(6):72. 10.1007/s00395-020-00827-7 33258071 PMC7704501

[39] Yang X An N Zhong C Guan M Jiang Y Zhang H Enhanced cardiomyocyte reactive oxygen species signaling promotes ibrutinib-induced atrial fibrillation. Redox Biol. (2020) 30:101432. 10.1016/j.redox.2020.101432 31986467 PMC6994714

[40] Ren M Li X Hao L Zhong J. Role of tumor necrosis factor alpha in the pathogenesis of atrial fibrillation: a novel potential therapeutic target? Ann Med. (2015) 47(4):316–24. 10.3109/07853890.2015.1042030 25982799

[41] Abe I Teshima Y Kondo H Kaku H Kira S Ikebe Y Association of fibrotic remodeling and cytokines/chemokines content in epicardial adipose tissue with atrial myocardial fibrosis in patients with atrial fibrillation. Heart Rhythm. (2018) 15(11):1717–27. 10.1016/j.hrthm.2018.06.025 29908372

[42] Cabaro S Conte M Moschetta D Petraglia L Valerio V Romano S Epicardial adipose tissue-derived IL-1β triggers postoperative atrial fibrillation. Front Cell Dev Biol. (2022) 10:893729. 10.3389/fcell.2022.893729 35721500 PMC9198900

[43] Liu T Li G. Periatrial epicardial fat, local pro- and anti-inflammatory balance, and atrial fibrillation. J Am Coll Cardiol. (2011) 57(10):1249; author reply 1249. 10.1016/j.jacc.2010.09.068 21371644

[44] Li X Garcia-Elias A Benito B Nattel S. The effects of cardiac stretch on atrial fibroblasts: analysis of the evidence and potential role in atrial fibrillation. Cardiovasc Res. (2022) 118(2):440–60. 10.1093/cvr/cvab035 33576384 PMC8803074

[45] Sohns C Marrouche NF. Atrial fibrillation and cardiac fibrosis. Eur Heart J. (2020) 41(10):1123–31. 10.1093/eurheartj/ehz786 31713590

[46] Boos CJ. Infection and atrial fibrillation: inflammation begets AF. Eur Heart J. (2020) 41(10):1120–2. 10.1093/eurheartj/ehz953 31971996

[47] Heijman J Muna AP Veleva T Molina CE Sutanto H Tekook M Atrial myocyte NLRP3/CaMKII nexus forms a substrate for postoperative atrial fibrillation. Circ Res. (2020) 127(8):1036–55. 10.1161/CIRCRESAHA.120.316710 32762493 PMC7604886

[48] Zhao J Zhang Y Yin Z Zhu Y Xin F Wang H. Impact of proinflammatory epicardial adipose tissue and differentially enhanced autonomic remodeling on human atrial fibrillation. J Thorac Cardiovasc Surg. (2023) 165(4):e158–74. 10.1016/j.jtcvs.2022.03.013 35461705

[49] Hanna P Buch E Stavrakis S Meyer C Tompkins JD Ardell JL Neuroscientific therapies for atrial fibrillation. Cardiovasc Res. (2021) 117(7):1732–45. 10.1093/cvr/cvab172 33989382 PMC8208752

[50] Capilupi MJ Kerath SM Becker LB. Vagus nerve stimulation and the cardiovascular system. Cold Spring Harb Perspect Med. (2020) 10(2):a034173. 10.1101/cshperspect.a034173 31109966 PMC6996447

[51] Andersen JH Andreasen L Olesen MS. Atrial fibrillation-a complex polygenetic disease. Eur J Hum Genet. (2021) 29(7):1051–60. 10.1038/s41431-020-00784-8 33279945 PMC8298566

[52] O’Sullivan JW Raghavan S Marquez-Luna C Luzum JA Damrauer SM Ashley EA Polygenic risk scores for cardiovascular disease: a scientific statement from the American Heart Association. Circulation. (2022) 146(8):e93–118. 10.1161/CIR.0000000000001077 35862132 PMC9847481

[53] Ajoolabady A Nattel S Lip GYH Ren J. Inflammasome signaling in atrial fibrillation: JACC state-of-the-art review. J Am Coll Cardiol. (2022) 79(23):2349–66. 10.1016/j.jacc.2022.03.379 35680186 PMC8972346

[54] Yao C Veleva T Scott L Cao S Chen G Jeyabal P Enhanced cardiomyocyte NLRP3 inflammasome signaling promotes atrial fibrillation. Circulation. (2018) 138(20):2227–42. 10.1161/CIRCULATIONAHA.118.035202 29802206 PMC6252285

[55] Zhang X Zhang Z Zhao Y Jiang N Qiu J Yang Y Alogliptin, a dipeptidyl peptidase-4 inhibitor, alleviates atrial remodeling and improves mitochondrial function and biogenesis in diabetic rabbits. J Am Heart Assoc. (2017) 6(5):e005945. 10.1161/JAHA.117.005945 28507060 PMC5524117

[56] Bi X Song Y Song Y Yuan J Cui J Zhao S Collagen cross-linking is associated with cardiac remodeling in hypertrophic obstructive cardiomyopathy. J Am Heart Assoc. (2021) 10(1):e017752. 10.1161/JAHA.120.017752 33356379 PMC7955480

[57] Chen S Yin L Xu Z Liu A-R Wang Y Yao W-B Inhibiting receptor for advanced glycation end product (AGE) and oxidative stress involved in the protective effect mediated by glucagon-like peptide-1 receptor on AGE induced neuronal apoptosis. Neurosci Lett. (2016) 612:193–8. 10.1016/j.neulet.2015.12.007 26679229

[58] Durak A Turan B. Liraglutide provides cardioprotection through the recovery of mitochondrial dysfunction and oxidative stress in aging hearts. J Physiol Biochem. (2023) 79(2):297–311. 10.1007/s13105-022-00939-9 36515811

[59] Bell DSH Goncalves E. Atrial fibrillation and type 2 diabetes: prevalence, etiology, pathophysiology and effect of anti-diabetic therapies. Diabetes Obes Metab. (2019) 21(2):210–7. 10.1111/dom.13512 30144274

[60] Saito S Teshima Y Fukui A Kondo H Nishio S Nakagawa M Glucose fluctuations increase the incidence of atrial fibrillation in diabetic rats. Cardiovasc Res. (2014) 104(1):5–14. 10.1093/cvr/cvu176 25082849

[61] Durak A Akkus E Canpolat AG Tuncay E Corapcioglu D Turan B. Glucagon-like peptide-1 receptor agonist treatment of high carbohydrate intake-induced metabolic syndrome provides pleiotropic effects on cardiac dysfunction through alleviations in electrical and intracellular Ca2+ abnormalities and mitochondrial dysfunction. Clin Exp Pharmacol Physiol. (2022) 49(1):46–59. 10.1111/1440-1681.13590 34519087

[62] Talman AH Psaltis PJ Cameron JD Meredith IT Seneviratne SK Wong DTL. Epicardial adipose tissue: far more than a fat depot. Cardiovasc Diagn Ther. (2014) 4(6):416–29. 10.3978/j.issn.2223-3652.2014.11.05 25610800 PMC4278038

[63] Iacobellis G. Epicardial adipose tissue in contemporary cardiology. Nat Rev Cardiol. (2022) 19(9):593–606. 10.1038/s41569-022-00679-9 35296869 PMC8926097

[64] Iacobellis G Willens HJ. Echocardiographic epicardial fat: a review of research and clinical applications. J Am Soc Echocardiogr. (2009) 22(12):1311–9; quiz 1417–18. 10.1016/j.echo.2009.10.013 19944955

[65] Iacobellis G Assael F Ribaudo MC Zappaterreno A Alessi G Di Mario U Epicardial fat from echocardiography: a new method for visceral adipose tissue prediction. Obes Res. (2003) 11(2):304–10. 10.1038/oby.2003.45 12582228

[66] Iacobellis G Ribaudo MC Assael F Vecci E Tiberti C Zappaterreno A Echocardiographic epicardial adipose tissue is related to anthropometric and clinical parameters of metabolic syndrome: a new indicator of cardiovascular risk. J Clin Endocrinol Metab. (2003) 88(11):5163–8. 10.1210/jc.2003-030698 14602744

[67] Gaborit B Sengenes C Ancel P Jacquier A Dutour A. Role of epicardial adipose tissue in health and disease: a matter of fat? Compr Physiol. (2017) 7(3):1051–82. 10.1002/cphy.c160034 28640452

[68] Rabkin SW. Epicardial fat: properties, function and relationship to obesity. Obes Rev. (2007) 8(3):253–61. 10.1111/j.1467-789X.2006.00293.x 17444966

[69] Burgeiro A Fuhrmann A Cherian S Espinoza D Jarak I Carvalho RA Glucose uptake and lipid metabolism are impaired in epicardial adipose tissue from heart failure patients with or without diabetes. Am J Physiol Endocrinol Metab. (2016) 310(7):E550–564. 10.1152/ajpendo.00384.2015 26814014 PMC4824138

[70] Aitken-Buck HM Moharram M Babakr AA Reijers R Van Hout I Fomison-Nurse IC Relationship between epicardial adipose tissue thickness and epicardial adipocyte size with increasing body mass index. Adipocyte. (2019) 8(1):412–20. 10.1080/21623945.2019.1701387 31829077 PMC6948959

[71] Aitken-Buck HM Babakr AA Coffey S Jones PP Tse RD Lamberts RR. Epicardial adipocyte size does not correlate with body mass index. Cardiovasc Pathol. (2019) 43:107144. 10.1016/j.carpath.2019.07.003 31491646

[72] Eiras S Teijeira-Fernández E Salgado-Somoza A Couso E García-Caballero T Sierra J Relationship between epicardial adipose tissue adipocyte size and MCP-1 expression. Cytokine. (2010) 51(2):207–12. 10.1016/j.cyto.2010.05.009 20610178

[73] Waddell HMM Moore MK Herbert-Olsen MA Stiles MK Tse RD Coffey S Identifying sex differences in predictors of epicardial fat cell morphology. Adipocyte. (2022) 11(1):325–34. 10.1080/21623945.2022.2073854 35531882 PMC9122305

[74] Bertaso AG Bertol D Duncan BB Foppa M. Epicardial fat: definition, measurements and systematic review of main outcomes. Arq Bras Cardiol. (2013) 101(1):e18–28. 10.5935/abc.20130138 23917514 PMC3998169

[75] Conte M Petraglia L Poggio P Valerio V Cabaro S Campana P Inflammation and cardiovascular diseases in the elderly: the role of epicardial adipose tissue. Front Med (Lausanne). (2022) 9:844266. 10.3389/fmed.2022.844266 35242789 PMC8887867

[76] Iacobellis G. Epicardial fat links obesity to cardiovascular diseases. Prog Cardiovasc Dis. (2023) 78:27–33. 10.1016/j.pcad.2023.04.006 37105279

[77] Vural B Atalar F Ciftci C Demirkan A Susleyici-Duman B Gunay D Presence of fatty-acid-binding protein 4 expression in human epicardial adipose tissue in metabolic syndrome. Cardiovasc Pathol. (2008) 17(6):392–8. 10.1016/j.carpath.2008.02.006 18417367

[78] Du Y Ji Q Cai L Huang F Lai Y Liu Y Association between omentin-1 expression in human epicardial adipose tissue and coronary atherosclerosis. Cardiovasc Diabetol. (2016) 15:90. 10.1186/s12933-016-0406-5 27352781 PMC4924240

[79] Wu L Dalal R Cao CD Postoak JL Yang G Zhang Q IL-10-producing B cells are enriched in murine pericardial adipose tissues and ameliorate the outcome of acute myocardial infarction. Proc Natl Acad Sci U S A. (2019) 116(43):21673–84. 10.1073/pnas.1911464116 31591231 PMC6815157

[80] Ji Q Zhang J Du Y Zhu E Wang Z Que B Human epicardial adipose tissue-derived and circulating secreted frizzled-related protein 4 (SFRP4) levels are increased in patients with coronary artery disease. Cardiovasc Diabetol. (2017) 16(1):133. 10.1186/s12933-017-0612-9 29037197 PMC5644066

[81] Gruzdeva O Uchasova E Dyleva Y Borodkina D Akbasheva O Belik E Relationships between epicardial adipose tissue thickness and adipo-fibrokine indicator profiles post-myocardial infarction. Cardiovasc Diabetol. (2018) 17(1):40. 10.1186/s12933-018-0679-y 29548286 PMC5855976

[82] van Rosendael AR Smit JM El’Mahdiui M Leung M Delgado V Bax JJ. Association between left atrial epicardial fat, left atrial volume, and the severity of atrial fibrillation. Europace. (2022) 24(8):1223–8. 10.1093/europace/euac031 35355079

[83] Sacks HS Fain JN Holman B Cheema P Chary A Parks F Uncoupling protein-1 and related messenger ribonucleic acids in human epicardial and other adipose tissues: epicardial fat functioning as brown fat. J Clin Endocrinol Metab. (2009) 94(9):3611–5. 10.1210/jc.2009-0571 19567523

[84] Christensen RH von Scholten BJ Hansen CS Jensen MT Vilsbøll T Rossing P Epicardial adipose tissue predicts incident cardiovascular disease and mortality in patients with type 2 diabetes. Cardiovasc Diabetol. (2019) 18(1):114. 10.1186/s12933-019-0917-y 31470858 PMC6716926

[85] Madonna R Massaro M Scoditti E Pescetelli I De Caterina R. The epicardial adipose tissue and the coronary arteries: dangerous liaisons. Cardiovasc Res. (2019) 115(6):1013–25. 10.1093/cvr/cvz062 30903194

[86] Conceição G Martins D M Miranda I Leite-Moreira AF Vitorino R Falcão-Pires I. Unraveling the role of epicardial adipose tissue in coronary artery disease: partners in crime? Int J Mol Sci. (2020) 21(22):8866. 10.3390/ijms21228866 33238643 PMC7700147

[87] Shaihov-Teper O Ram E Ballan N Brzezinski RY Naftali-Shani N Masoud R Extracellular vesicles from epicardial fat facilitate atrial fibrillation. Circulation. (2021) 143(25):2475–93. 10.1161/CIRCULATIONAHA.120.052009 33793321

[88] Echols JT Wang S Patel AR Hogwood AC Abbate A Epstein FH. Fatty acid composition MRI of epicardial adipose tissue: methods and detection of proinflammatory biomarkers in ST-segment elevation myocardial infarction patients. Magn Reson Med. (2025) 93(2):519–35. 10.1002/mrm.30285 39323040 PMC11604849

[89] Antonopoulos AS Sanna F Sabharwal N Thomas S Oikonomou EK Herdman L Detecting human coronary inflammation by imaging perivascular fat. Sci Transl Med. (2017) 9(398):eaal2658. 10.1126/scitranslmed.aal2658 28701474

[90] Nogajski Ł Mazuruk M Kacperska M Kurpias M Mączewski M Nowakowski M Epicardial fat density obtained with computed tomography imaging—more important than volume? Cardiovasc Diabetol. (2024) 23(1):389. 10.1186/s12933-024-02474-x 39472958 PMC11523889

[91] West HW Siddique M Williams MC Volpe L Desai R Lyasheva M Deep-learning for epicardial adipose tissue assessment with computed tomography: implications for cardiovascular risk prediction. JACC Cardiovasc Imaging. (2023) 16(6):800–16. 10.1016/j.jcmg.2022.11.018 36881425 PMC10663979

[92] Bekkers SCAM Backes WH Kim RJ Snoep G Gorgels APM Passos VL Detection and characteristics of microvascular obstruction in reperfused acute myocardial infarction using an optimized protocol for contrast-enhanced cardiovascular magnetic resonance imaging. Eur Radiol. (2009) 19(12):2904–12. 10.1007/s00330-009-1489-0 19588152 PMC2778783

[93] Ng ACT Strudwick M van der Geest RJ Gillinder L Goo SY Cowin G Impact of epicardial adipose tissue, left ventricular myocardial fat content, and interstitial fibrosis on myocardial Contractile function. Circ Cardiovasc Imaging. (2018) 11(8):e007372. 10.1161/CIRCIMAGING.117.007372 30354491

[94] Henningsson M Brundin M Scheffel T Edin C Viola F Carlhäll CJ. Quantification of epicardial fat using 3D cine Dixon MRI. BMC Med Imaging. (2020) 20(1):80. 10.1186/s12880-020-00478-z 32664848 PMC7362508

[95] Iacobellis G Baroni MG. Cardiovascular risk reduction throughout GLP-1 receptor agonist and SGLT2 inhibitor modulation of epicardial fat. J Endocrinol Invest. (2022) 45(3):489–95. 10.1007/s40618-021-01687-1 34643917

[96] Huber AT Fankhauser S Wittmer S Chollet L Lam A Maurhofer J Epicardial adipose tissue dispersion at CT and recurrent atrial fibrillation after pulmonary vein isolation. Eur Radiol. (2024) 34(8):4928–38. 10.1007/s00330-023-10498-2 38197916 PMC11255050

[97] Yamaguchi Y Cavallero S Patterson M Shen H Xu J Kumar SR Adipogenesis and epicardial adipose tissue: a novel fate of the epicardium induced by mesenchymal transformation and PPAR*γ* activation. Proc Natl Acad Sci U S A. (2015) 112(7):2070–5. 10.1073/pnas.1417232112 25646471 PMC4343131

[98] Suffee N Moore-Morris T Farahmand P Rücker-Martin C Dilanian G Fradet M Atrial natriuretic peptide regulates adipose tissue accumulation in adult atria. Proc Natl Acad Sci U S A. (2017) 114(5):E771–80. 10.1073/pnas.1610968114 28096344 PMC5293064

[99] Zhao J Cheng W Dai Y Li Y Feng Y Tan Y Excessive accumulation of epicardial adipose tissue promotes microvascular obstruction formation after myocardial ischemia/reperfusion through modulating macrophages polarization. Cardiovasc Diabetol. (2024) 23(1):236. 10.1186/s12933-024-02342-8 38970123 PMC11227217

[100] Gaborit B Venteclef N Ancel P Pelloux V Gariboldi V Leprince P Human epicardial adipose tissue has a specific transcriptomic signature depending on its anatomical peri-atrial, peri-ventricular, or peri-coronary location. Cardiovasc Res. (2015) 108(1):62–73. 10.1093/cvr/cvv208 26239655

[101] Shah R Patel T Freedman JE. Circulating extracellular vesicles in human disease. N Engl J Med. (2018) 379(10):958–66. 10.1056/NEJMra1704286 30184457

[102] Sluijter JPG Davidson SM Boulanger CM Buzás EI de Kleijn DPV Engel FB Extracellular vesicles in diagnostics and therapy of the ischaemic heart: position paper from the working group on cellular biology of the heart of the European Society of Cardiology. Cardiovasc Res. (2018) 114(1):19–34. 10.1093/cvr/cvx211 29106545 PMC5852624

[103] Wang Q Xi W Yin L Shen H Gao Y Min J Human epicardial adipose tissue cTGF expression is an independent risk factor for atrial fibrillation and highly associated with atrial fibrosis. Sci Rep. (2018) 8(1):3585. 10.1038/s41598-018-21911-y 29483593 PMC5827202

[104] Kita S Maeda N Shimomura I. Interorgan communication by exosomes, adipose tissue, and adiponectin in metabolic syndrome. J Clin Invest. (2019) 129(10):4041–9. 10.1172/JCI129193 31483293 PMC6763291

[105] Allessie MA de Groot NMS Houben RPM Schotten U Boersma E Smeets JL Electropathological substrate of long-standing persistent atrial fibrillation in patients with structural heart disease: longitudinal dissociation. Circ Arrhythm Electrophysiol. (2010) 3(6):606–15. 10.1161/CIRCEP.109.910125 20719881

[106] Hu YF Chen YJ Lin YJ Chen SA. Inflammation and the pathogenesis of atrial fibrillation. Nat Rev Cardiol. (2015) 12(4):230–43. 10.1038/nrcardio.2015.2 25622848

[107] Igarashi T Finet JE Takeuchi A Fujino Y Strom M Greener ID Connexin gene transfer preserves conduction velocity and prevents atrial fibrillation. Circulation. (2012) 125(2):216–25. 10.1161/CIRCULATIONAHA.111.053272 22158756 PMC3260348

[108] Iacobellis G Zaki MC Garcia D Willens HJ. Epicardial fat in atrial fibrillation and heart failure. Horm Metab Res. (2014) 46(8):587–90. 10.1055/s-0034-1367078 24557503

[109] Szekeres Z Nagy A Jahner K Szabados E. Impact of selected glucagon-like peptide-1 receptor agonists on Serum lipids, adipose tissue, and muscle metabolism-A narrative review. Int J Mol Sci. (2024) 25(15):8214. 10.3390/ijms25158214 39125786 PMC11311305

[110] Lee J Umana IE Nguyen J. Exacerbation of atrial fibrillation related to dulaglutide use. Clin Case Rep. (2021) 9(5):e04223. 10.1002/ccr3.4223 34026191 PMC8123549

[111] Hamed K Alosaimi MN Ali BA Alghamdi A Alkhashi T Alkhaldi SS Glucagon-Like peptide-1 (GLP-1) receptor agonists: exploring their impact on diabetes, obesity, and cardiovascular health through a comprehensive literature review. Cureus. (2024) 16(9):e68390. 10.7759/cureus.68390 39355484 PMC11444311

[112] Zhao L Zhu C Lu M Chen C Nie X Abudukerimu B The key role of a glucagon-like peptide-1 receptor agonist in body fat redistribution. J Endocrinol. (2019) 240(2):271–86. 10.1530/JOE-18-0374 30530905

[113] McLean BA Wong CK Kabir MG Drucker DJ. Glucagon-like peptide-1 receptor Tie2+ cells are essential for the cardioprotective actions of liraglutide in mice with experimental myocardial infarction. Mol Metab. (2022) 66:101641. 10.1016/j.molmet.2022.101641 36396031 PMC9706177

[114] Wei Y Mojsov S. Distribution of GLP-1 and PACAP receptors in human tissues. Acta Physiol Scand. (1996) 157(3):355–7. 10.1046/j.1365-201X.1996.42256000.x 8830893

[115] Zheng Z Zong Y Ma Y Tian Y Pang Y Zhang C Glucagon-like peptide-1 receptor: mechanisms and advances in therapy. Signal Transduct Target Ther. (2024) 9(1):234. 10.1038/s41392-024-01931-z 39289339 PMC11408715

[116] Zhong J Chen H Liu Q Zhou S Liu Z Xiao Y. GLP-1 receptor agonists and myocardial metabolism in atrial fibrillation. J Pharm Anal. (2024) 14(5):100917. 10.1016/j.jpha.2023.12.007 38799233 PMC11127228

[117] Heuvelman VD Van Raalte DH Smits MM. Cardiovascular effects of glucagon-like peptide 1 receptor agonists: from mechanistic studies in humans to clinical outcomes. Cardiovasc Res. (2020) 116(5):916–30. 10.1093/cvr/cvz323 31825468

[118] Deng Y Li L Li Q Guo J Cai B Zhou F Central obesity as a potential causal risk factor for atrial fibrillation: evidence from Mendelian randomization study. Europace. (2024) 26(3):euae061. 10.1093/europace/euae061 38450558 PMC10951967

[119] Scheen AJ. Antidiabetic agents and risk of atrial fibrillation/flutter: a comparative critical analysis with a focus on differences between SGLT2 inhibitors and GLP-1 receptor agonists. Diabetes Metab. (2022) 48(6):101390. 10.1016/j.diabet.2022.101390 36170946

[120] Stoll L Lo JC. GLP-1 Receptor agonists, the holy grail preventing atrial fibrillation in patients with T2D? JACC Basic Transl Sci. (2023) 8(8):937–8. 10.1016/j.jacbts.2023.03.022 37719426 PMC10504427

[121] Wang J Guo R Ma X Zhang Q Zheng N Zhang J. Liraglutide inhibits AngII-induced cardiac fibroblast proliferation and ECM deposition through regulating miR-21/PTEN/PI3K pathway. Cell Tissue Bank. (2023) 24(1):125–37. 10.1007/s10561-022-10021-9 35792987

[122] Nakamura H Niwano S Niwano H Fukaya H Murakami M Kishihara J Liraglutide suppresses atrial electrophysiological changes. Heart Vessels. (2019) 34(8):1389–93. 10.1007/s00380-018-01327-4 30762094

[123] Xu Y Boyle TA Lyu B Ballew SH Selvin E Chang AR Glucagon-like peptide-1 receptor agonists and the risk of atrial fibrillation in adults with diabetes: a real-world study. J Gen Intern Med. (2024) 39(7):1112–21. 10.1007/s11606-023-08589-3 38191976 PMC11116290

[124] Zhang X Peng N Zhang X Zhu Z Miao Y Wu Y Association of glucagon-like peptide-1 receptor agonists with atrial fibrillation, cardiac arrest, and ventricular fibrillation: casual evidence from a drug target Mendelian randomization. Diabetol Metab Syndr. (2025) 17(1):179. 10.1186/s13098-025-01712-w 40442813 PMC12123724

[125] Saglietto A Falasconi G Penela D Francia P Sau A Ng FS Glucagon-like peptide-1 receptor agonist semaglutide reduces atrial fibrillation incidence: a systematic review and meta-analysis. Eur J Clin Invest. (2024) 54(12):e14292. 10.1111/eci.14292 39058274

[126] Zhang HD Ding L Liu K Mi L-J Yu F-Y Yan X-X Semaglutide for the prevention of atrial fibrillation: a systematic review and meta-analysis. Diabetes Metab Syndr. (2024) 18(6):103067. 10.1016/j.dsx.2024.103067 38955095

[127] Cesaro A Pastori D Acerbo V Biccirè FG Golino M Panico D Reduction of new onset of atrial fibrillation in patients treated with semaglutide: an updated systematic review and meta regression analysis of randomized controlled trials. Eur J Prev Cardiol. (2025):zwaf257. 10.1093/eurjpc/zwaf257 40294206

[128] Karakasis P Fragakis N Patoulias D Theofilis P Kassimis G Karamitsos T Effects of glucagon-like peptide 1 receptor agonists on atrial fibrillation recurrence after catheter ablation: a systematic review and meta-analysis. Adv Ther. (2024) 41(10):3749–56. 10.1007/s12325-024-02959-x 39141282

[129] Monami M Nreu B Scatena A Giannini S Andreozzi F Sesti G Glucagon-like peptide-1 receptor agonists and atrial fibrillation: a systematic review and meta-analysis of randomised controlled trials. J Endocrinol Invest. (2017) 40(11):1251–8. 10.1007/s40618-017-0698-7 28569363

[130] Fisher M Petrie MC Ambery PD Donaldson J Ye J McMurray JJV. Cardiovascular safety of albiglutide in the harmony programme: a meta-analysis. Lancet Diabetes Endocrinol. (2015) 3(9):697–703. 10.1016/S2213-8587(15)00233-8 26276240

[131] Shi W Zhang W Zhang D Ren G Wang P Gao L Comparison of the effect of glucose-lowering agents on the risk of atrial fibrillation: a network meta-analysis. Heart Rhythm. (2021) 18(7):1090–6. 10.1016/j.hrthm.2021.03.007 33684547

[132] Fauchier G Bisson A Bodin A Herbert J Angoulvant D Ducluzeau PH Glucose-lowering drug use and new-onset atrial fibrillation in patients with diabetes mellitus. Diabetologia. (2021) 64(11):2602–5. 10.1007/s00125-021-05551-y 34435218

[133] Hamedi Z Mishriky BM Okunrintemi V Powell JR Cummings DM. GLP-1 RA and atrial fibrillation in the cardiovascular outcome trials. Diabetes Metab Res Rev. (2021) 37(5):e3436. 10.1002/dmrr.3436 33440044

[134] Wu S Lu W Chen Z Dai Y Chen K Zhang S. Association of glucagon-like peptide-1 receptor agonists with cardiac arrhythmias in patients with type 2 diabetes or obesity: a systematic review and meta-analysis of randomized controlled trials. Diabetol Metab Syndr. (2022) 14(1):195. 10.1186/s13098-022-00970-2 36572913 PMC9791739

[135] Krychtiuk KA Marquis-Gravel G Murphy S Chiswell K Green JB Leiter LA Albiglutide and atrial fibrillation in patients with type 2 diabetes and established cardiovascular disease—insights from the harmony outcomes trial. Eur J Prev Cardiol. (2026) 33(1):30–41. 10.1093/eurjpc/zwae379 39602568

[136] Raubenheimer PJ Cushman WC Avezum A Basile J Conget I Dagenais G Dulaglutide and incident atrial fibrillation or flutter in patients with type 2 diabetes: a *post hoc* analysis from the REWIND randomized trial. Diabetes Obes Metab. (2022) 24(4):704–12. 10.1111/dom.14634 34984808

[137] Satti DI Karius A Chan JSK Isakadze N Yadav R Garg K Effects of glucagon-like peptide-1 receptor agonists on atrial fibrillation recurrence after catheter ablation. JACC Clin Electrophysiol. (2024) 10(8):1848–55. 10.1016/j.jacep.2024.03.031 38795099

[138] Karakasis P Patoulias D Tzeis S Fragakis N. Glucagon-Like peptide-1 receptor agonists and atrial fibrillation recurrence after ablation: a fire without the smoke? JACC Clin Electrophysiol. (2024) 10(8):1940–1. 10.1016/j.jacep.2024.07.004 39197973

[139] Wu JY Tseng KJ Kao CL Hung KC Yu T Lin YM. Clinical effectiveness of tirzepatide for patients with atrial fibrillation and type 2 diabetes: a retrospective cohort study. Diabetes Res Clin Pract. (2025) 225:112279. 10.1016/j.diabres.2025.112279 40412626

[140] Glaser K Glaser W Marino L Ruchala M Bilotta F. Impact of glucagon-like peptide-1 receptor agonists on the incidence of atrial fibrillation. World J Cardiol. (2025) 17(7):107510. 10.4330/wjc.v17.i7.107510 40741027 PMC12304861

[141] Guo J Song Z Wang S Yao D Hong Y Fu M Effect of semaglutide on atrial arrhythmias recurrence following ablation for atrial fibrillation: a prospective study. Circ Arrhythm Electrophysiol. (2025) 18(11):e014069. 10.1161/CIRCEP.125.014069 41064855

[142] Venier S Defaye P Lochon L Benali R Bisson A Carabelli A Impact of GLP-1 receptor agonist therapy on atrial fibrillation recurrence after catheter ablation in obese patients: a real-world data analysis. Circ Arrhythm Electrophysiol. (2026) 19(1):e014101. 10.1161/CIRCEP.125.014101 41446932 PMC12822759

[143] Chan CS Lin FJ Chen YC Higa S. Glucagon-like peptide-1 receptor activation reduces pulmonary vein arrhythmogenesis and regulates calcium homeostasis. Int J Mol Sci. (2023) 24(17):13100. 10.3390/ijms241713100 37685906 PMC10488086

[144] Zhou Q Hao G Xie W Chen B Lu W Wang G Exenatide reduces atrial fibrillation susceptibility by inhibiting hKv1.5 and hNav1.5 channels. J Biol Chem. (2024) 300(5):107294. 10.1016/j.jbc.2024.107294 38636665 PMC11109313

[145] Dai Y He H Li S Yang L Wang X Liu Z. Comparison of the efficacy of glucagon-like peptide-1 receptor agonists in patients with metabolic associated fatty liver disease: updated systematic review and meta-analysis. Front Endocrinol (Lausanne. (2020) 11:622589. 10.3389/fendo.2020.622589 33664710 PMC7924308

[146] Robinson LE Holt TA Rees K Randeva HS O’Hare JP. Effects of exenatide and liraglutide on heart rate, blood pressure and body weight: systematic review and meta-analysis. BMJ Open. (2013) 3(1):e001986. 10.1136/bmjopen-2012-001986 23355666 PMC3563145

[147] Lubberding AF Veedfald S Achter JS Nissen SD Soattin L Sorrentino A Glucagon-like peptide-1 increases heart rate by a direct action on the sinus node. Cardiovasc Res. (2024) 120(12):1427–41. 10.1093/cvr/cvae120 38832935 PMC11472427

[148] Li W Chen X Xie X Xu M Xu L Liu P Comparison of sodium-glucose cotransporter 2 inhibitors and glucagon-like peptide receptor agonists for atrial fibrillation in type 2 diabetes Mellitus: systematic review with network meta-analysis of randomized controlled trials. J Cardiovasc Pharmacol. (2022) 79(3):281–8. 10.1097/FJC.0000000000001197 34935705

[149] Chen J Xu S Wang L Zhou W Deng N Tang Q Exendin-4 inhibits atrial arrhythmogenesis in a model of myocardial infarction-induced heart failure via the GLP-1 receptor signaling pathway. Exp Ther Med. (2020) 20(4):3669–78. 10.3892/etm.2020.9089 32855719 PMC7444344

[150] Cheng Y Liu P Xiang Q Liang J Zhang H Yang L. Glucagon-like peptide-1 attenuates diabetes-associated osteoporosis in ZDF rat, possibly through the RAGE pathway. BMC Musculoskelet Disord. (2022) 23(1):465. 10.1186/s12891-022-05396-5 35581617 PMC9112483

[151] Chen J Xu S Zhou W Wu L Wang L Li W. Exendin-4 reduces ventricular arrhythmia activity and calcium sparks-mediated sarcoplasmic reticulum Ca leak in rats with heart failure. Int Heart J. (2020) 61(1):145–52. 10.1536/ihj.19-327 31956148

[152] Younce CW Burmeister MA Ayala JE. Exendin-4 attenuates high glucose-induced cardiomyocyte apoptosis via inhibition of endoplasmic reticulum stress and activation of SERCA2a. Am J Physiol Cell Physiol. (2013) 304(6):C508–18. 10.1152/ajpcell.00248.2012 23302777

[153] Huang JH Chen YC Lee TI Kao Y-H Chazo T-F. Glucagon-like peptide-1 regulates calcium homeostasis and electrophysiological activities of HL-1 cardiomyocytes. Peptides. (2016) 78:91–8. 10.1016/j.peptides.2016.02.007 26930508

[154] Nattel S Burstein B Dobrev D. Atrial remodeling and atrial fibrillation: mechanisms and implications. Circ Arrhythm Electrophysiol. (2008) 1(1):62–73. 10.1161/CIRCEP.107.754564 19808395

[155] Chen YJ Chen SA Chang MS Lin CI. Arrhythmogenic activity of cardiac muscle in pulmonary veins of the dog: implication for the genesis of atrial fibrillation. Cardiovasc Res. (2000) 48(2):265–73. 10.1016/s0008-6363(00)00179-6 11054473

[156] Puglisi S Rossini A Poli R Dughera F Pia A Terzolo M Effects of SGLT2 inhibitors and GLP-1 receptor agonists on renin-angiotensin-aldosterone system. Front Endocrinol (Lausanne. (2021) 12:738848. 10.3389/fendo.2021.738848 34745006 PMC8567993

[157] Luna-Marco C Iannantuoni F Hermo-Argibay A Devos D Salazar JD Víctor VM Cardiovascular benefits of SGLT2 inhibitors and GLP-1 receptor agonists through effects on mitochondrial function and oxidative stress. Free Radic Biol Med. (2024) 213:19–35. 10.1016/j.freeradbiomed.2024.01.015 38220031

[158] Batta A Hatwal J Sharma YP. Assessment of coronary artery disease in non-valvular atrial fibrillation: is this light at the End of the tunnel? Vasc Health Risk Manag. (2024) 20:493–9. 10.2147/VHRM.S484638 39534246 PMC11556227

[159] Batta A Hatwal J Batta A Verma S Sharma YP. Atrial fibrillation and coronary artery disease: an integrative review focusing on therapeutic implications of this relationship. World J Cardiol. (2023) 15(5):229–43. 10.4330/wjc.v15.i5.229 37274376 PMC10237004

[160] Ussher JR Drucker DJ. Cardiovascular actions of incretin-based therapies. Circ Res. (2014) 114(11):1788–803. 10.1161/CIRCRESAHA.114.301958 24855202

[161] Dozio E Vianello E Malavazos AE Tacchini L Schmitz G Iacobellis G Epicardial adipose tissue GLP-1 receptor is associated with genes involved in fatty acid oxidation and white-to-brown fat differentiation: a target to modulate cardiovascular risk? Int J Cardiol. (2019) 292:218–24. 10.1016/j.ijcard.2019.04.039 31023563

[162] Iacobellis G Villasante Fricke AC. Effects of semaglutide versus dulaglutide on epicardial fat thickness in subjects with type 2 diabetes and obesity. J Endocr Soc. (2020) 4(4):bvz042. 10.1210/jendso/bvz042 32190806 PMC7069837

[163] Li Y Liu X Li G Zhang P. Effect of liraglutide on epicardial adipose tissue thickness with echocardiography in patients with obese type 2 diabetes mellitus. Int J Diabetes Dev Ctries. (2020) 40(4):500–6. 10.1007/s13410-020-00820-9

[164] Dutour A Abdesselam I Ancel P Kober F Mrad G Darmon P Exenatide decreases liver fat content and epicardial adipose tissue in patients with obesity and type 2 diabetes: a prospective randomized clinical trial using magnetic resonance imaging and spectroscopy. Diabetes Obes Metab. (2016) 18(9):882–91. 10.1111/dom.12680 27106272

[165] Myasoedova VA Parisi V Moschetta D Valerio V Conte M Massaiu I Efficacy of cardiometabolic drugs in reduction of epicardial adipose tissue: a systematic review and meta-analysis. Cardiovasc Diabetol. (2023) 22(1):23. 10.1186/s12933-023-01738-2 36721184 PMC9890718

[166] Richard D Picard F. Brown fat biology and thermogenesis. Front Biosci (Landmark Ed). (2011) 16(4):1233–60. 10.2741/3786 21196229

[167] Vendrell J El Bekay R Peral B García-Fuentes E Megia A Macias-Gonzalez M Study of the potential association of adipose tissue GLP-1 receptor with obesity and insulin resistance. Endocrinology. (2011) 152(11):4072–9. 10.1210/en.2011-1070 21862620

[168] Yang J Ren J Song J Liu F Wu C Wang X Glucagon-like peptide 1 regulates adipogenesis in 3T3-L1 preadipocytes. Int J Mol Med. (2013) 31(6):1429–35. 10.3892/ijmm.2013.1350 23588664

[169] van Eyk HJ Paiman EHM Bizino MB de Heer P Geelhoed-Duijvestijn PH Kharagjitsingh AV A double-blind, placebo-controlled, randomised trial to assess the effect of liraglutide on ectopic fat accumulation in south Asian type 2 diabetes patients. Cardiovasc Diabetol. (2019) 18(1):87. 10.1186/s12933-019-0890-5 31288820 PMC6615254

[170] Bizino MB Jazet IM de Heer P van Eyk HJ Dekkers IA Rensen PCN Placebo-controlled randomised trial with liraglutide on magnetic resonance endpoints in individuals with type 2 diabetes: a pre-specified secondary study on ectopic fat accumulation. Diabetologia. (2020) 63(1):65–74. 10.1007/s00125-019-05021-6 31690988 PMC6890592

[171] He F Chen W Xu W Liu D Xiao Z Tang Y Safety and efficacy of liraglutide on reducing visceral and ectopic fat in adults with or without type 2 diabetes mellitus: a systematic review and meta-analysis. Diabetes Obes Metab. (2023) 25(3):664–74. 10.1111/dom.14908 36314246

